# Alternative splicing dynamics and evolutionary divergence during embryogenesis in wheat species

**DOI:** 10.1111/pbi.13579

**Published:** 2021-03-24

**Authors:** Peng Gao, Teagen D. Quilichini, Chun Zhai, Li Qin, Kirby T. Nilsen, Qiang Li, Andrew G. Sharpe, Leon V. Kochian, Jitao Zou, Anireddy S.N. Reddy, Yangdou Wei, Curtis Pozniak, Nii Patterson, C. Stewart Gillmor, Raju Datla, Daoquan Xiang

**Affiliations:** ^1^ Global Institute for Food Security University of Saskatchewan Saskatoon SK Canada; ^2^ Aquatic and Crop Resource Development National Research Council Canada Saskatoon SK Canada; ^3^ Agriculture and Agri‐Food Canada Saskatoon Research and Development Centre Saskatoon SK Canada; ^4^ College of Art & Science University of Saskatchewan Saskatoon SK Canada; ^5^ Agriculture and Agri‐Food Canada Brandon Research and Development Centre Brandon MB Canada; ^6^ National Key Laboratory of Crop Genetic Improvement Huazhong Agricultural University Wuhan China; ^7^ Department of Biology and Program in Cell and Molecular Biology Colorado State University Fort Collins CO USA; ^8^ Crop Development Centre University of Saskatchewan Saskatoon SK Canada; ^9^ Laboratorio Nacional de Genómica para la Biodiversidad (Langebio) Unidad de Genómica Avanzada Centro de Investigación y Estudios Avanzados del IPN (CINVESTAV‐IPN) Irapuato Guanajuato Mexico

**Keywords:** alternative splicing, polyploid, evolution, embryogenesis, wheat, grain development

## Abstract

Among polyploid species with complex genomic architecture, variations in the regulation of alternative splicing (AS) provide opportunities for transcriptional and proteomic plasticity and the potential for generating trait diversities. However, the evolution of AS and its influence on grain development in diploid grass and valuable polyploid wheat crops are poorly understood. To address this knowledge gap, we developed a pipeline for the analysis of alternatively spliced transcript isoforms, which takes the high sequence similarity among polyploid wheat subgenomes into account. Through analysis of synteny and detection of collinearity of homoeologous subgenomes, conserved and specific AS events across five wheat and grass species were identified. A global analysis of the regulation of AS in diploid grass and polyploid wheat grains revealed diversity in AS events not only between the endosperm, pericarp and embryo overdevelopment, but also between subgenomes. Analysis of AS in homoeologous triads of polyploid wheats revealed evolutionary divergence between gene‐level and transcript‐level regulation of embryogenesis. Evolutionary age analysis indicated that the generation of novel transcript isoforms has occurred in young genes at a more rapid rate than in ancient genes. These findings, together with the development of comprehensive AS resources for wheat and grass species, advance understanding of the evolution of regulatory features of AS during embryogenesis and grain development in wheat.

## Introduction

Through evolutionary time, genomes and the genes they contain are ever‐changing (Long *et al.,* 2016). Polyploidization events enable significant genomic change and have occurred repeatedly over the course of plant evolution in numerous crop species including wheat (Adams and Wendel, 2005). In the global staple wheat crop of the *Poaceae* family, the primary species used in grain production are hexaploid *Triticum (T.) aestivum* and tetraploid *Triticum turgidum* ssp. *durum*, which putatively arose through the hybridization and polyploidization of three diploid grass ancestors, *Triticum urartu*, *Aegilops (Ae.) speltoides* and *Aegilops tauschii* (Otto, 2007; Ramírez‐González *et al.,* 2018). The genomic expansion produced by polyploidization events can alter gene expression, splicing events and methylation, providing the potential for phenotypic change and diversity. Changes in gene expression (GE) and alternative splicing (AS) represent major sources of species‐specific divergence (Calarco *et al.,* 2007), and their spatiotemporal regulations are essential for diverse plant developmental processes such as embryogenesis, seed formation and flowering time induction (Filichkin *et al.,* 2010; Gao *et al.,* 2019; Koltunow *et al.,* 1990; Thatcher *et al.,* 2016; Wang *et al.,* 2014; Xiang *et al.,* 2019).

Alternative splicing, or the varied processing of a gene's mRNA transcript, results in the translation of multiple proteins with different amino acid sequences from a single gene (Reddy, 2001). AS of multi‐exon precursor mRNAs (pre‐mRNAs) increases gene coding potential and expands protein structural complexity, allowing diversification of gene function (Hartmann *et al.,* 2016; Reddy, 2007; Reddy *et al.,* 2013; Staiger and Brown, 2013). In addition to contributing to proteome diversity, AS can generate truncated proteins with regulatory potential or toxicity (Chaudhary *et al.,* 2019; Neverov *et al.,* 2005; Nilsen and Graveley, 2010; Reddy, 2007). AS also modulates gene expression through the production of isoforms that are degraded by nonsense‐mediated decay (Barbazuk *et al.,* 2008; Shang *et al.,* 2017; Staiger and Brown, 2013). In plants, AS is a widespread phenomenon, with 33%–60% of genes undergoing AS (Brett *et al.,* 2002; Chen *et al.,* 2018; Hartmann *et al.,* 2016; Mazzucotelli *et al.,* 2008; Mei *et al.,* 2017; Staiger and Brown, 2013; Thatcher *et al.,* 2016). AS has been studied in numerous economically valued grass species, such as wheat (*T. aestivum*), rice (*Oryza sativa*), maize (*Zea mays*), cotton (*Gossypium hirsutum*) and barley (*Hordeum vulgare*) (Chen *et al.,* 2018; Liu *et al.,* 2018; Mandadi and Scholthof, 2015; Mei *et al.,* 2017; Wang *et al.,* 2018b; Zhang *et al.,* 2019).

Closely related species can exhibit tremendous morphological diversity, despite high genome sequence similarity (Erwin, 2007; Minelli, 2016). Wheat grains display diverse phenotypic characteristics, yet share similar repertoires of subgenomes between diploid and polyploid species (Dubcovsky and Dvorak, 2007). In wheat, grain development is determined by transcriptional, post‐transcriptional and post‐translational networks, which include as a key component the AS of thousands of genes (Ramírez‐González *et al.,* 2018). Furthermore, as a result of polyploidization, most genes in tetraploid and hexaploid wheat species are present in multiple copies, referred to as ‘homoeologs’, among the A, B and D subgenomes (Devos, 2010; Glover *et al.,* 2016; Krasileva *et al.,* 2017). Recent studies of the developing grains of ancestral diploid grasses and polyploid wheats revealed dynamic RNA expression and considerable evolutionary diversity, likely underpinning many of the physiological differences in grain traits (Ramírez‐González *et al.,* 2018; Xiang *et al.,* 2019). However, the occurrence and evolution of AS in diploid and polyploid wheat genomes and the regulation of AS in grain development have not been characterized. Studies on the landscape of AS in multiple tissues of vertebrate species have revealed evolutionary divergence of gene and isoform regulation (Barbosa‐Morais *et al.,* 2012; Merkin *et al.,* 2012), indicating different trajectories for the evolution of genes and transcripts under the pressures of natural selection. The evolutionary context provided by studies on AS in ancestral diploid grass species and their polyploid wheat derivatives offers the potential to reveal divergence in transcriptional and post‐transcriptional regulation among species and cultivars subjected to domestication, breeding and selection. In the present study, Illumina RNA‐seq was used to catalog the diversity of mRNA AS in select ancestral grass and wheat species. Using a pipeline for alternative transcript isoform analysis that addresses the high sequence similarity in polyploid species, we explored the regulation of AS in embryo, endosperm and pericarp development of hexaploid wheats, tetraploid wheats and their putative diploid grass ancestors. This study provides valuable new insights into the evolution of AS during embryogenesis and grain development in wheat species, uncovering bias between gene‐level and transcript‐level regulation.

## Results

### RNA‐seq profiling of developing grain tissues for a comprehensive analysis of AS in wheats and ancestral grasses

**Figure 1:**
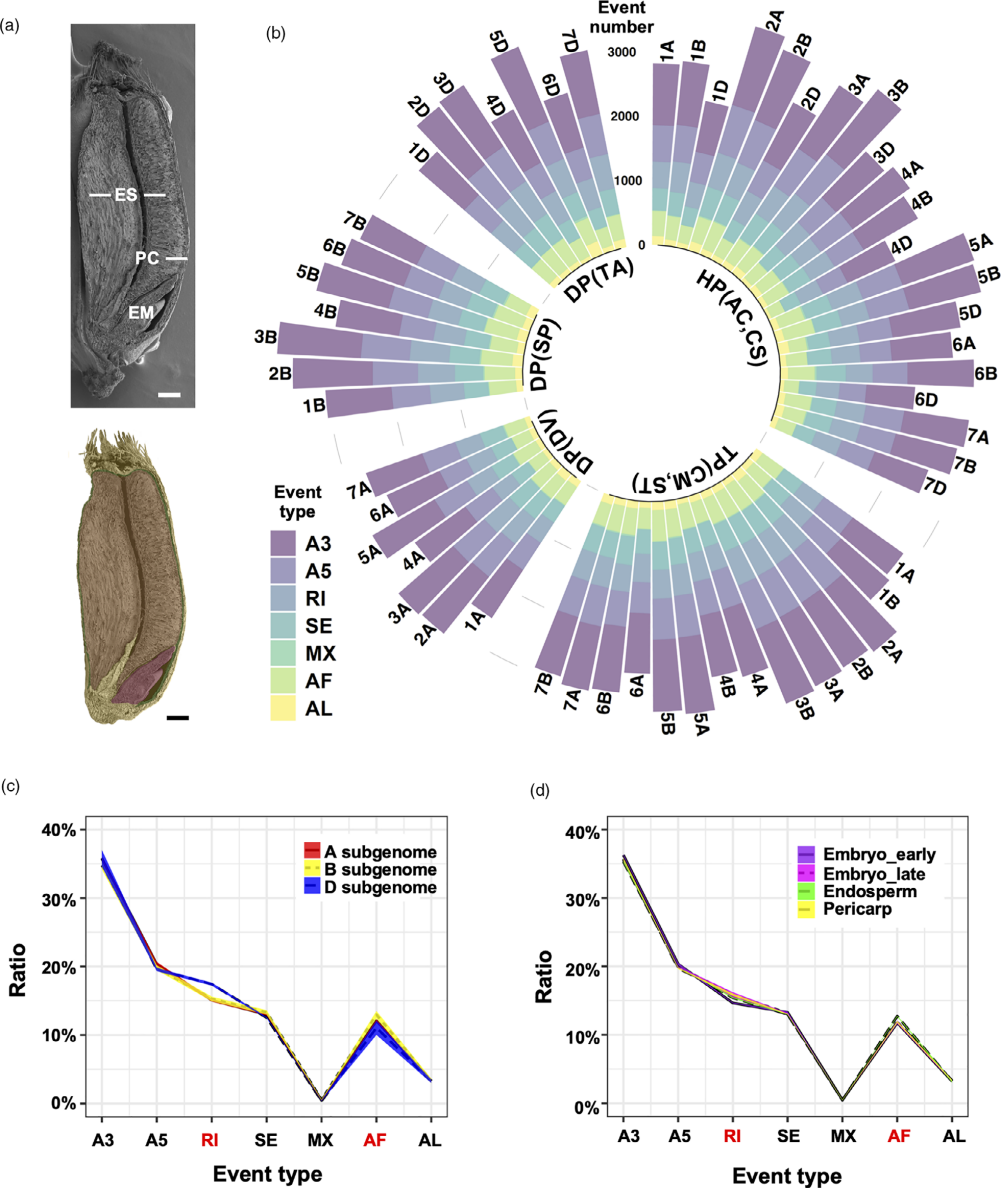
AS profiling of different wheat varieties. (a) Scanning electron micrograph of an AC Barrie grain at E05 stage, longitudinally cut and false‐coloured to reveal the embryo (EM), endosperm (ES) and pericarp (PC). Three main components of the grain are false‐coloured with pink representing embryo, brown representing endosperm and green representing pericarp. Bar = 5 mm. (b) AS event distribution at the chromosome level. Each bar is labelled with a chromosome name (at the end of each bar), colour‐coded by AS event type (see inset), and clustered with the corresponding species and ploidy level. HP, hexaploid; TP, tetraploid; DP, diploid; AC, AC Barrie; CS, Chinese Spring; CM, CDC Commander; ST, CDC Strong Field; DV, *Triticum monococcum* DV92; SP, *Aegilops speltoides* TA2780; TA, *Aegilops tauschii* TA101132. (c) Proportion of each AS type in different subgenomes and (d) at different stages/tissues. *X*‐axis, seven types of AS events with red texts indicating a significant difference among pairwise comparisons (Student's *t*‐test, *P*‐value < 0.001). *Y*‐axis, the AS event type proportions in total events. Filled regions indicate standard deviations between varieties. Embryo_early, E01–E03; Embryo_late, E04–E07; Endosperm, E08–E09; Pericarp, E10.

To examine the contribution of mRNA splicing to the evolution of the wheat grain, we isolated three grain components: the embryo, endosperm and pericarp (Figure 1a) from a tetraploid (AABB, *T. durum* CDC Commander) and a hexaploid (AABBDD, *T. aestivum* Chinese Spring) wheat species, and performed deep‐sequencing of samples from sequential stages of grain development (defined as E01–E10, see Methods). In addition to the tetraploid and hexaploid wheats studied here, we previously isolated these key subcompartments from the developing grain of five related species of polyploid wheats and their putative diploid ancestral relatives, and analysed their transcriptomes using the Illumina Hi‐Seq platform (Xiang *et al.,* 2019). By combining these transcriptome data sets, we obtained a comprehensive data set for investigating the AS landscape during polyploidization and the evolution of wheat grain development. Altogether, this study examines 140 RNA‐seq libraries comprising five related wheat and grass species (consisting of seven varieties) across seven developmental stages and three tissues of the grain, generating 8.2 billion paired‐end reads (Table [Link], [Link]). This in‐depth coverage of transcriptomes of developing grain tissues in related polyploid wheat and diploid grass species provided evolutionary context for the global analysis of AS events in grain formation.

Polyploid wheats show high sequence similarity among subgenome homoeologs, making it difficult to distinguish and accurately assign splicing isoforms to their respective homoeologs. Therefore, we developed a comprehensive AS pipeline to explore transcriptome complexity in polyploid wheats, including the three subgenomes (A, B and D) of hexaploid wheat, at the transcript isoform level (see Methods and Figure [Link], [Link]). This bioinformatics pipeline comprises three phases of analysis: (i) library preparation, reads preprocessing, alignment and assembly; (ii) transcript quantification; and (iii) downstream splicing analysis including events profiling, percent spliced‐in (PSI) calculation and evolutionary analysis. Taken together, the development of a novel pipeline for AS detection in polyploids enabled clear distinction of subgenome differences at the gene‐ and transcript‐level to be captured (Figure [Link], [Link]).

### Isoform detection for a global view of AS events during grain development

The reliable detection of expressed exons, splicing junctions, and distinction of the origin of each subgenome isoform are pre‐requisites for the comprehensive characterization of AS in polyploids. In the hexaploid (AABBDD, Chinese Spring‐CS and AC Barrie‐AC), tetraploid (AABB, CDC Commander‐CM and CDC Strong field‐ST) and three diploid species analysed with our pipeline, including *Triticum monococcum* (AA, DV92‐DV), *Ae. speltoides* (BB, TA2780‐SP) and *Ae. tauschii* (DD, TA101132‐TA), we identified 223 873 (AABBDD); 154 535 (AABB); 75 691 (AA); 78 844 (BB) and 69 338 (DD) nonredundant splice isoforms and captured 44 205 (AABBDD); 30 122 (AABB); 14 711 (AA); 15 411 (BB) and 14 083 (DD) multi‐transcript loci, with transcripts per locus ranging from 2.1 to 2.3 (Table [Link], [Link]). Of the genes subject to AS, 46% exhibited one additional AS isoform, while approximately 10% had more than five AS events (Table [Link], [Link]).

Seven types of AS events were defined: alternative 5′ splice site (A5), alternative 3′ splice site (A3), retained intron (RI), skipped exon (SE), mutually exclusive exons (MX), alternative first exon (AF) and alternative last exon (AL) (Figure [Link], [Link]). For the five species examined, A3 was the most abundant type of AS and represented 33% of the AS events detected, followed by A5 (19%), RI (16%), SE (13%), AF (13%), AL (5%) and MX (0.6%) (Figure [Link], [Link]). The distribution of AS events by type was defined at the subgenome, chromosome, stage and tissue levels (Figure 1b, Figure [Link], [Link]). Among the five species' subgenomes, the B subgenome of the diploid SP had the highest number of AS events, and the D subgenome of hexaploid wheat species (AC and CS) had the lowest number of AS events (Figure 1b). Chromosomes 1D, 4D and 6D possessed significantly fewer AS events than other chromosomes, while chromosomes 2A, 2B, 3A and 3B were enriched for AS in all species examined (Duncan's multiple range test – DMRT). The spatial distribution of AS events detected in the grain revealed enrichment in the embryo over the endosperm and pericarp in corresponding stages of development, with fewer events in early stages of embryo development (E01–E03) than in late stages (E04–E07) (Figure [Link], [Link]B).

To investigate the spatiotemporal distribution of AS events, the proportion of AS event types across all stages of grain development, the three tissues of the grain, and the A, B and D subgenomes was calculated (Figure 1c,d). The D subgenome exhibited significant differences from the A and B subgenomes in the total number of AS events, with a higher proportion of RI (Student's *t*‐test, *P*‐value < 0.05), indicating the potential for a greater evolutionary distance of the D subgenome from the A and B subgenomes, at the level of isoform formation (Figure 1c). This pattern of reduced AS events in the D subgenome is consistent with a previous analysis of AS in the leaf and root of wheat (Yu *et al.,* 2020), suggesting conservation of this trend across the plant body parts. In grain tissue comparisons, a lower proportion of RI was detected in the early‐stage embryo than in the endosperm, pericarp and late‐stage embryo, and a higher proportion of AF events were identified in the endosperm (Figure 1d). These results reveal variation in the distribution of AS event types over wheat grain development and highlight select types of AS (e.g. RI) as putative key players in the evolution of wheat grain tissues.

**Figure 2:**
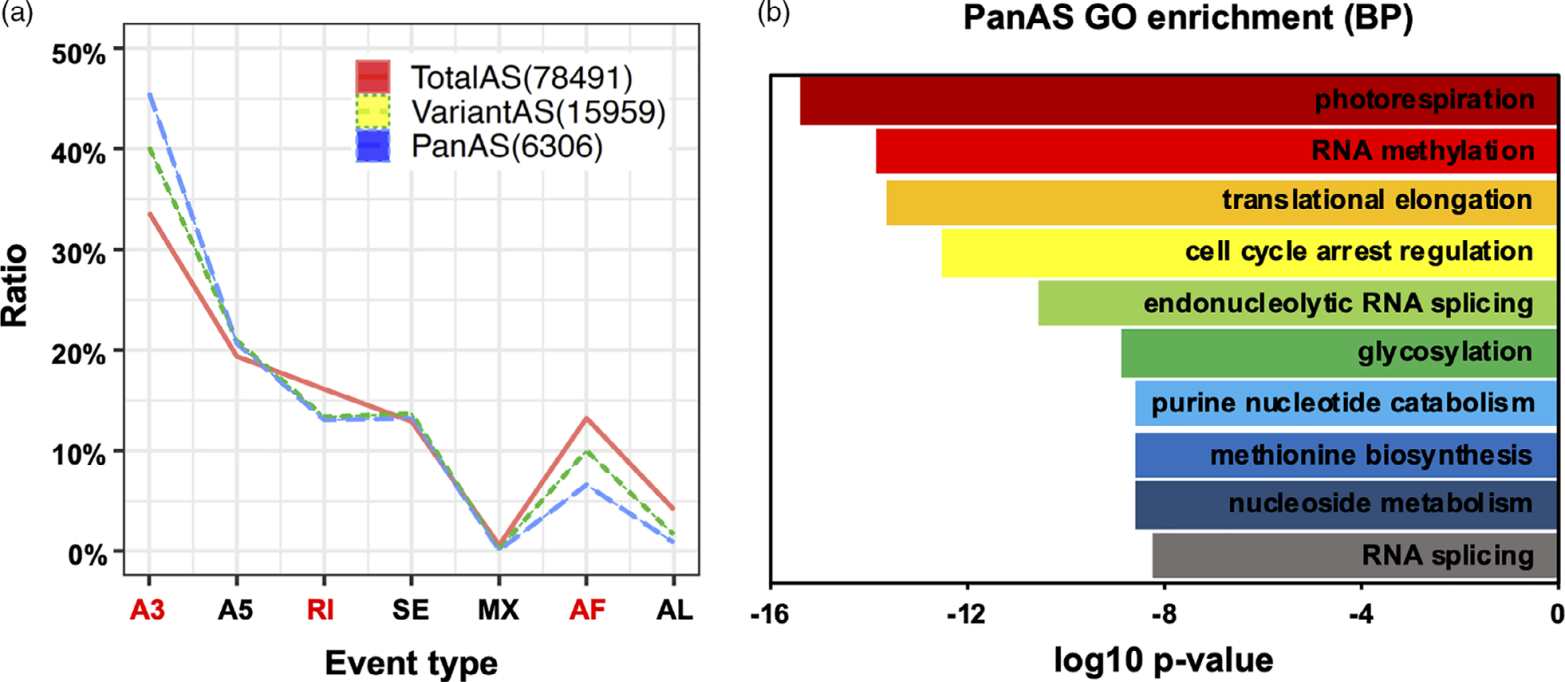
Analysis of a subgroup of AS events (PanAS) that are alternatively spliced in most wheat embryos. (a) The proportion of each AS event type in total events from different event subgroups. *X*‐axis, seven types of AS events; red labels indicate a significant difference among pairwise comparison. *Y*‐axis, the AS event type proportions in total events. (b) The top ten significantly enriched Gene Ontology (GO) terms and corresponding p‐values from a GO enrichment analysis of PanAS events in the biological process (BP) category.

Percent inclusion or percent spliced‐in (PSI), defined as the efficiency of splicing a specific exon into the transcript population of a gene, has been widely used to evaluate splice junction sites (Barbosa‐Morais *et al.,* 2012). RNA‐seq reads were mapped to the junction database to determine PSI, and exons with 5 ≤ PSI ≤ 95 were considered reliable AS events for a gene. A list of all AS types and PSI values was generated for each of the five species (Data [Link], [Link], [Link], [Link], [Link], [Link], [Link], [Link]). To identify different biological aspects of grain development, we divided AS events into subgroups based on PSI values. One AS subgroup containing AS events that are active (5 < PSI < 95) in >80% of the samples with sufficient read coverage was termed as ‘PanAS’ (Tapial *et al.,* 2017). Another AS subgroup called ‘VariantAS’, contained AS events with a PSI difference of 25 (PSI_max_ − PSI_min_ > 25). The distribution of AS events across species, developmental stages and grain tissues, including comparisons among PanAS, VariantAS and the total number of AS events (TotalAS) is presented, with 6306 PanAS and 15 959 VariantAS events identified in the five species (Figure 2a, Data [Link], [Link]). The A3, RI and AF types of AS displayed notable differences in PanAS, VariantAS and TotalAS, with a lower proportion of RI and AF and a higher proportion of A3 events in PanAS and VariantAS subgroups relative to the TotalAS (Student's *t*‐test, *P*‐value < 0.05), suggesting that AS event type bias exists.

When identifying AS events in plants, distinguishing functional AS from background noise is challenging. One way to validate a spliced isoform is to examine whether it is conserved across evolutionarily related species (Mei *et al.,* 2017), which belongs to PanAS in this study. To gain insight into the function of conserved AS events, we performed Gene Ontology (GO) enrichment analysis of all genes in the PanAS subgroup. The top enriched GO terms in the biological process (BP) category were photorespiration, RNA methylation, translational elongation, cell cycle arrest regulation and endonucleolytic RNA splicing (Figure 2b). Since AS events in the PanAS subgroup were identified in most samples of this study, these GO terms represent conserved biological processes during grain formation in polyploid wheats and their putative diploid ancestors.

### Evolutionary variation between alternative splicing and gene expression

**Figure 3:**
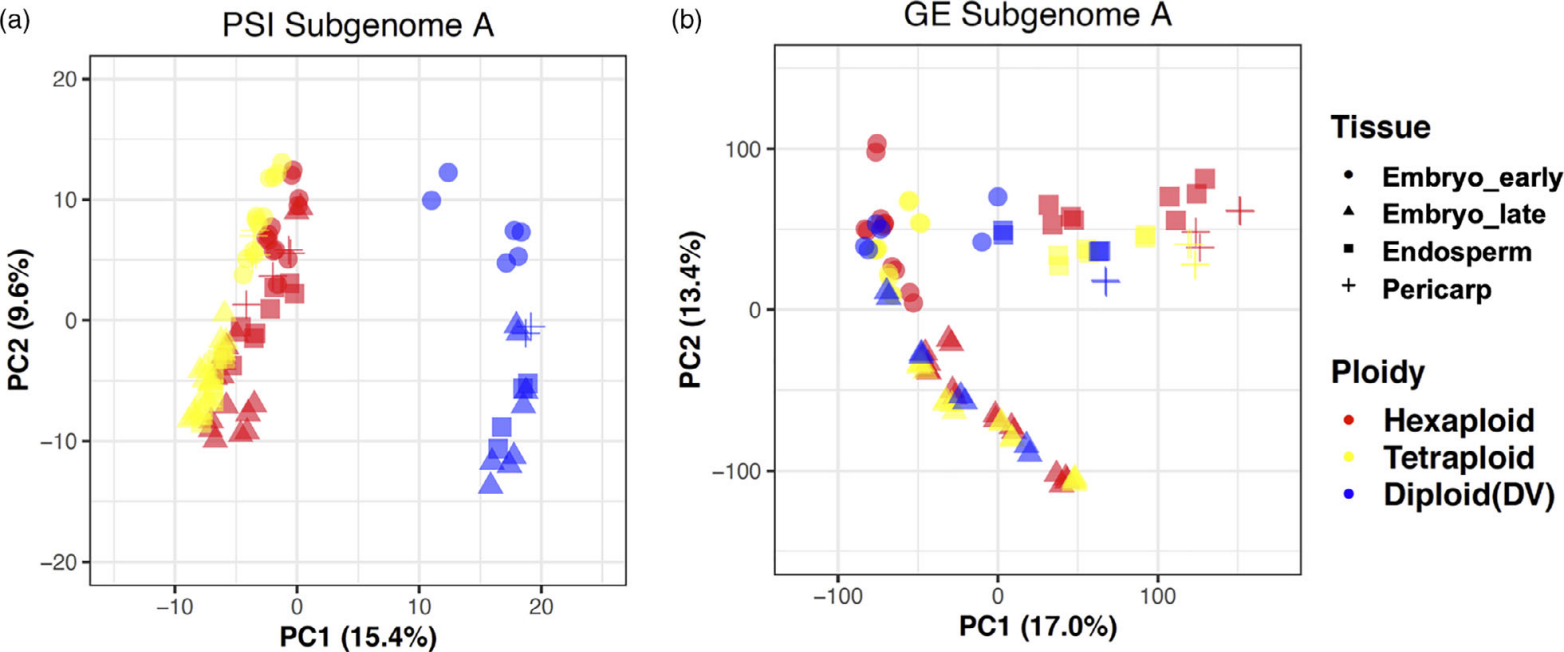
Principal component analysis (PCA) of gene expression and alternative splicing regulation in the A subgenome of select wheat and grass species. PCA based on PSI values (a) or based on gene expression (GE) (b) of five lines from wheat and ancestral grass species. *X*‐axis and *Y*‐axis indicate two principal components (PC), PC1 and PC2, respectively. The grain tissues, including early and late stages of embryo, are labelled with different shapes, and ploidy levels are colour‐coded (see insets). The fraction of variance explained by each principal component is indicated in the following bracket. Embryo_early, E01–E03; Embryo_late, E04–E07; Endosperm, E08–E09; Pericarp, E10. Hexaploid, AC Barrie (AC) and Chinese Spring (CS); Tetraploid, CDC Commander (CM) and CDC Strong Field (ST); Diploid (DV), *Triticum monococcum* DV92.

To assess AS and its evolutionary role in the development of the embryo, endosperm and pericarp of wheat grains, we performed principal component analysis (PCA) using PSI values of PanAS events for diploid, tetraploid and hexaploid wheats. Gene expression (GE) data for genes containing PanAS events were used to perform an independent PCA, for comparison. The resulting PCA data allowed pairwise comparison between AS and GE at the subgenome level, for the identification of putative regulatory differences between genes and transcript isoforms. Three pairwise PCA analyses based on the A, B and D subgenomes for different species, stages and tissues displayed similar segregation patterns (Figure 3, Figure [Link], [Link]A–D, Table [Link], [Link]). AS profiles, on the basis of overall PSI value correlation, revealed the separation of samples first by ploidy, followed by stage of development and tissue (Figure 3a, Figure [Link], [Link]A,C). Especially, the diploid grasses showed clear separation from tetraploid and hexaploid wheats. In contrast, PCA based on GE segregated samples first by stage and tissue, and then ploidy, with some exceptions for the B subgenome (Figure 3b, Figure [Link], [Link]B,D).

Since tetraploid and hexaploid wheats clustered close together in principle component 1 (PC1) of the AS PCA, an additional analysis utilizing only polyploid species data was performed. For the A and B subgenomes, AS and GE segregation patterns were compared through PCA (Figure [Link], [Link]E,F). Developmental stage was again the primary source of variability for GE segregation patterns. However, for the PCA of AS, the stage and tissue variations separated in PC1, while ploidy variations separated PC2. This result is different from the PCA separation containing diploid species, highlighting the potential for unique AS regulation mechanisms between diploid, tetraploid and hexaploid wheat species. In all PCA plots, samples from the same stage or tissue were separated by longer distances in AS than in GE, indicating that stage‐ or tissue‐specific AS has evolved at a more rapid rate than stage‐ or tissue‐specific GE.

PCA is an unsupervised dimensionality reduction technique used to identify correlations and patterns. To validate our PCA and examine the relationship between AS and GE in polyploid wheats and their diploid ancestors, we performed a supervised clustering analysis using partial least squares discriminant analysis (PLS‐DA) on the same data sets. In the PLS‐DA analysis, forced separation of developmental stages and tissues for both AS and GE yielded similar results to the PCA (Figures S5, S6). Forced separation of grain development stages and tissues for each of the subgenomes, produced clear clustering of GE samples into stages and tissues (Figure [Link], [Link]A,C,E), and a clustering pattern that did not emerge for AS samples (Figure [Link], [Link]B,D,F). Instead, clustering by ploidy largely persisted for AS samples with the stage/tissue‐forced model. As shown in B and D subgenome comparisons (Figure [Link], [Link]D,F), AS in diploid species (SP and TA) was tightly clustered and separated from their respective polyploid progenies, providing further evidence supporting the strength of ploidy for explaining AS variance.

### Identification of specific AS patterns

**Figure 4:**
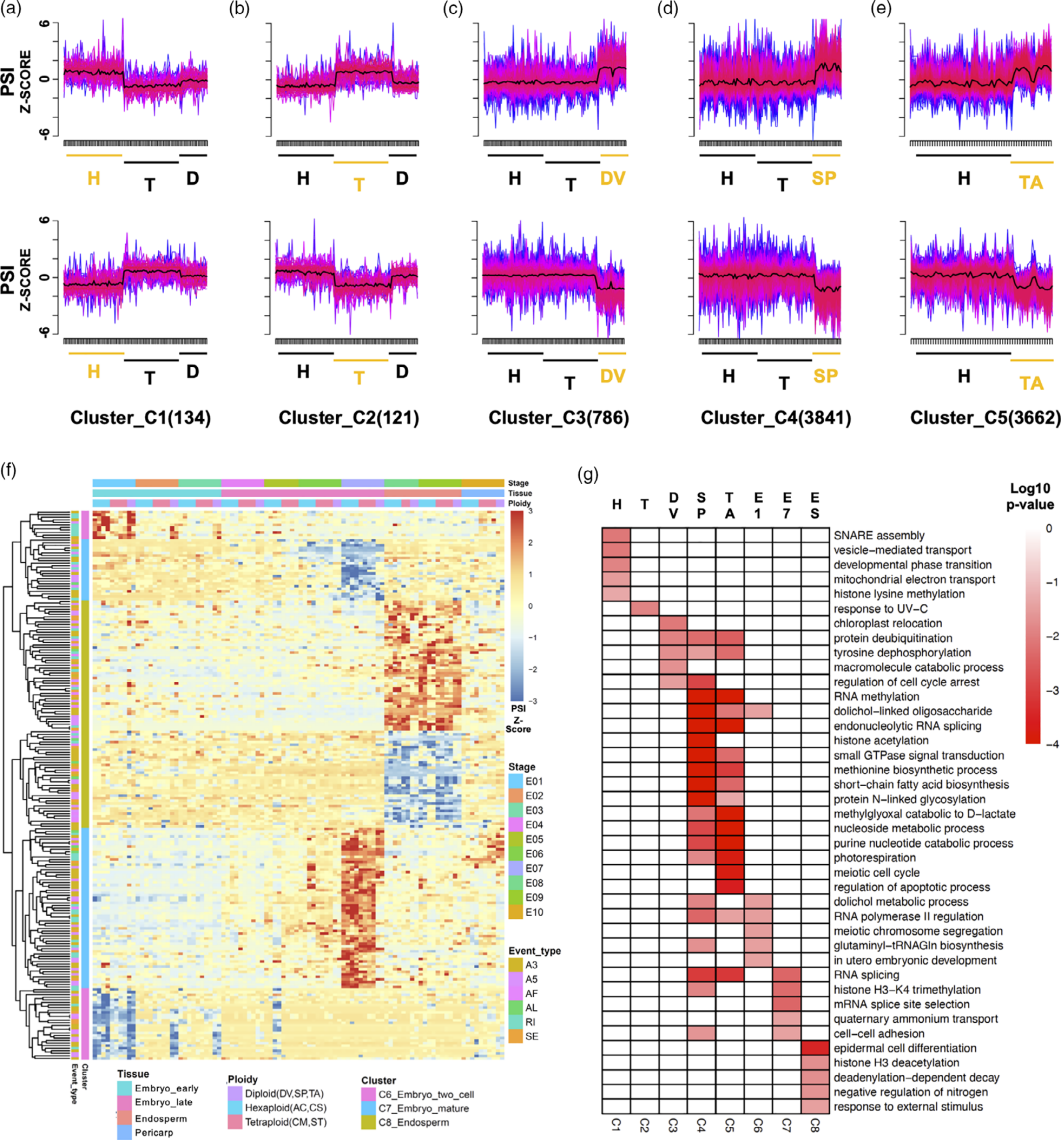
Characterization of specific AS events across five wheat and grass species. (a–e) *k*‐mean clusters of ploidy‐specific events in hexaploid (a) and tetraploid (b) wheats, and grass diploids DV (c), SP (d) and TA (e). Clusters with significantly different PSI values for a specific ploidy are generated based on significantly higher (top) and lower (bottom) PSI values for each ploidy. The specific ploidy in each cluster are labelled in orange below the *X*‐axis of each chart. The number of ploidy‐specific events for each cluster is indicated in brackets, following each cluster name. *X*‐axis, individual embryo samples from different stages, tissues and species, subdivided by ploidy level. H, hexaploid (AC, CS); T, tetraploid (CM, ST); D, Diploid, consisting of three species: DV, SP and TA. *Y*‐axis, Z‐score normalized PSI value. Centres of clusters (Centroids) are indicated by a black line. The correlation between AS events and the cluster centres are indicated by coloured lines, from red, to pink, to purple, to blue (high to low). (f) Heat map of normalized PSI value from stage‐specific (E01, E07) and endosperm‐specific AS events (E08, E09). (g) GO enrichment, in the biological process category, of transcripts from each AS event cluster; the top five significantly enriched GO terms were selected for plotting, E1, two cell embryo‐specific; E7, mature embryo‐specific; ES, endosperm‐specific. C1‐C8, Cluster_C1 to Cluster_C8 shown in a–f.

From the clustering analyses, ploidy emerged as the major source of AS pattern variation among wheat and ancestral grass species. To investigate the effect of ploidy on AS, we extracted diploid‐, tetraploid‐ and hexaploid‐specific AS events from our AS data sets. *K*‐mean clustering using PSI values was performed to group similar AS events and identify AS clustering amongst varying ploidy levels through correlation analysis (Figure 4a–e, Figure [Link], [Link]). For the A, B and D subgenomes, 24, 14 and 9 clusters were identified, respectively (Figure [Link], [Link], Data [Link], [Link]). Clusters positively or negatively correlated with each ploidy level were identified as ploidy‐specific AS event clusters (Figure 4a–e), with 134 hexaploid‐specific, 121 tetraploid‐specific, 786 DV, 3841 SP and 3662 TA diploid‐specific AS events recognized. Distribution analysis of ploidy‐specific AS events by type showed notable variance in A3, A5 and SE, while RI remained stable in all ploidy‐specific AS events (Figure [Link], [Link]), supporting conservation of RI during grain evolution.

*K*‐mean clustering of PSI values was then performed to identify stage‐ and tissue‐specific AS events, and presented in a heat map based on *Z*‐score transformed PSI values (Figure 4f, Data [Link], [Link]). Stage‐specific AS event clusters were uniquely identified in E01 and E07, with more E07‐specific AS events putatively indicating more complex splicing regulation in this later stage of embryonic development. E01‐specific AS events were found primarily in the hexaploid and diploid species examined, while the E07‐specific AS events were predominantly from the hexaploid and tetraploid species. For the threshold selected, tissue‐specific AS event clusters were only identified for the endosperm and were highly conserved across all species and ploidy levels (Figure 4f). These data support conserved splicing regulation of key biological process(es) that may be unique or enriched in the endosperm, such as the accumulation of starch and storage protein from the middle stages of grain development.

The inclusion of more than one cultivar of *T. aestivum*, as well as for the tetraploid species *T. durum* enabled intra‐species variations in AS to be explored. In CS and AC cultivar comparisons, 151 AS events that differ within this hexaploid species were identified (Data [Link], [Link]). In CM and ST cultivar comparisons, 36 AS events that differed between these tetraploid *T. durum* cultivars were identified (Data [Link], [Link]). The identification of cultivar‐specific AS events verifies the existence of isoform variation within a species and represents a useful resource for future studies aimed at uncovering the impact of splicing regulation differences on yield traits in polyploid wheat species.

The genes in the tissue‐specific, stage‐specific and ploidy‐specific AS clusters (named C1–C8) identified were examined using GO annotation and enrichment analysis, to gain insight into their biological relevance (Figure 4g, Data [Link], [Link]). From the ploidy‐specific data, AS events in hexaploids were significantly enriched in vesicle‐mediated transport, developmental phase transition and mitochondrial electron transport processes, while the protein deubiquitination and tyrosine dephosphorylation processes were enriched in the three diploid species, suggesting biased regulation in different ploidies during grain development. In pairwise comparisons of the three diploid species, SP (BB genome) and TA (DD genome) AS events shared the most GO terms, suggesting a close relationship between these species at the transcript level. Among the stage‐specific AS events, E01 GO terms were enriched in meiotic chromosome segregation, glutaminyl‐tRNA^Gln^ biosynthesis and utero embryonic development. E07‐specific AS GO terms were enriched in RNA splicing, mRNA splicing site selection and cell–cell adhesion (Figure 4g, Figure [Link], [Link]B). In grain tissue comparisons, the endosperm was characterized by AS events involved in deadenylation‐dependent decay, the negative regulation of nitrogen and in response to external stimulus processes. Interestingly, the enriched GO terms of tissue‐, stage‐ and ploidy‐specific AS were highly divergent from the corresponding specific GO terms at the gene‐level identified in Xiang *et al.,* 2019. This lack of significant overlap in dominant GO terminology suggests unique roles for GE and AS in regulating grain development. Taken together, AS appears to regulate grain development spatiotemporally and adds another layer of complexity to the mechanisms of transcriptional regulation operating in the grains of polyploid wheats and their putative ancestors.

### Dynamics of AS triads during grain development in polyploid wheats

In wheat, homoeologs with a 1:1:1 correspondence across the three homoeologous subgenomes are defined as triads (Ramírez‐González *et al.,* 2018). Although gene triads in hexaploid wheat have been identified, conserved AS triads have not been well documented. In this study, pairwise analysis among the A, B and D subgenomes based on the IWGSC RefSeq v1.0 assembly was performed to identify conserved junctions. The analysis was restricted to perfect 1:1 orthology relationships between exons for any given pair of subgenomes, and three chain files with chain format (https://genome.ucsc.edu/goldenPath/help/chain.html) including pairwise genomic coordinate conversions among the three genomes were generated (see Methods). This is the first time chain files have been built in hexaploid wheat, and can be used for comparative AS analyses and for corresponding SNP/Indel recognition among the three homoeologous subgenomes. Based on the three chain files, all AS events were utilized to select 1:1:1 overlaps in the three subgenomes as AS triads, which identified a total of 1950 conserved splicing events (Data [Link], [Link]).

**Figure 5:**
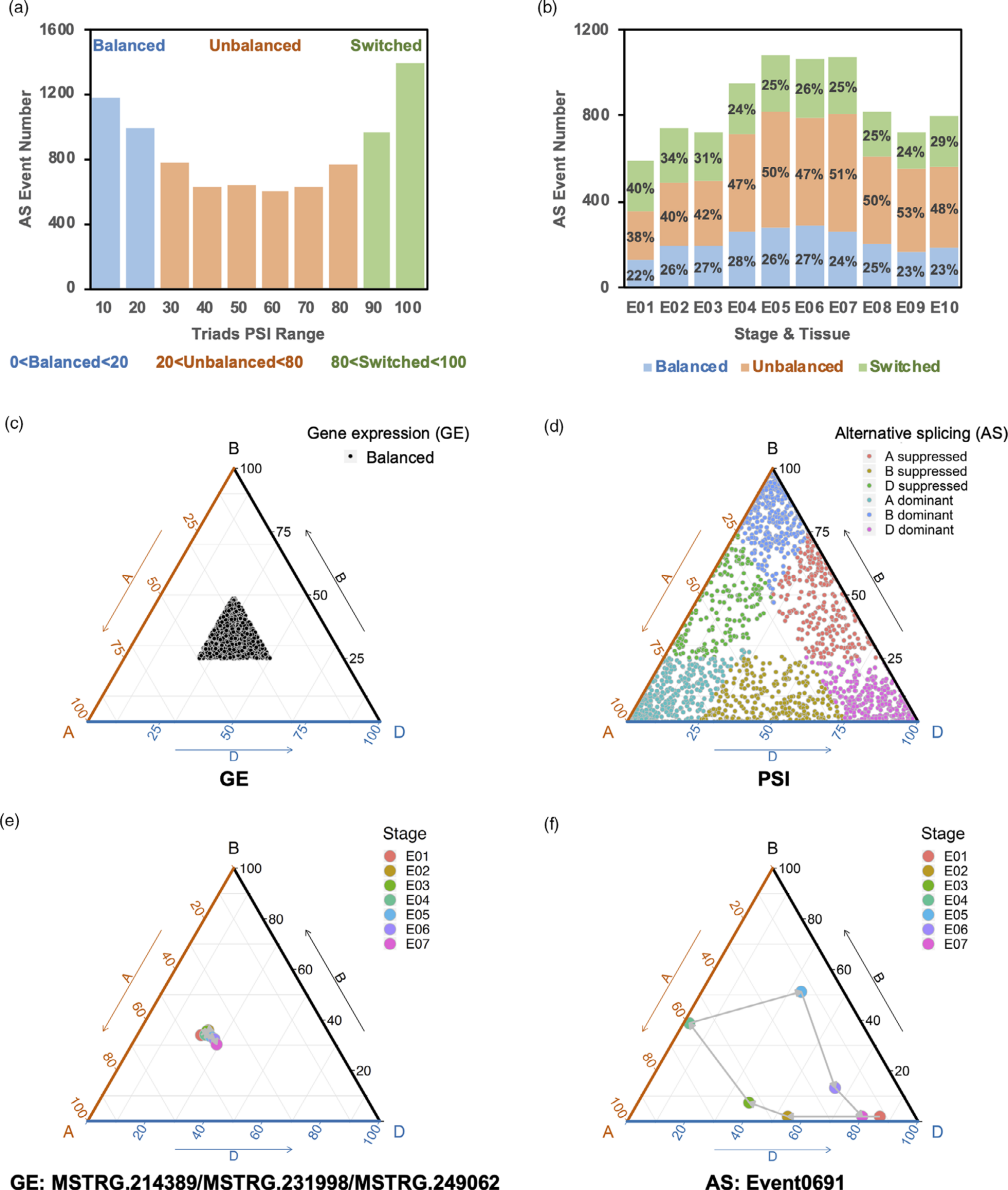
Characterization of conserved AS triads in the A, B, and D subgenomes of five wheat and grass species. (a) AS event numbers for three categories of conserved AS triads. The three categories of conserved AS triads, balanced (blue), unbalanced (orange) and switched (green), are defined by PSI ranges between 0 and 20, between 20 and 80 and between 80 and 100, as indicated. *X*‐axis, PSI from 0 (left) to 100 (right), grouped by tens. (b) Distribution of conserved AS triads, by category, across the ten stages and tissues (E01–E10) of embryogenesis. AS event ratios for each category are indicated as a percentage. (c–d) Ternplots based on gene expression (GE) (c) and PSI (d) values of select triad AS events in the unbalanced and switched categories in which gene expression levels are balanced among the three homoeologs from the A, B and D subgenomes. In (d), AS event triads from different groups are colour‐coded, as detailed in the inset. (e) Ternplot based on corresponding gene expression (TPM) of the AS triad Event0691, for the seven stages of developing embryo (E01–E07). (f) Ternplot based on the PSI values of three AS events from AS triad Event0691. The sequential embryonic developmental process from E01 to E07 is indicated by arrows. In all ternplots, three axes indicate A, B and D subgenomes, respectively. E01, two cell embryo; E02, pre‐embryo; E03, transition stage embryo; E04, leaf early‐stage embryo; E05, leaf middle stage embryo; E06, leaf late‐stage embryo; E07, mature embryo; E08, the transition stage endosperm; E09 leaf late‐stage endosperm; and E10, leaf early‐stage pericarp.

By comparing the PSI values difference (defined as the difference between the maximum and minimum PSI values from an AS triad) for each AS triad among the three subgenomes across grain development and between grain tissues, splicing bias was calculated and AS triads were categorized into one of three groups: (i) Balanced, in which PSI values for the three homoeologs of a triad are similar (with a PSI range of 0–20) and splicing bias is low; (ii) Unbalanced, in which PSI values for the three homoeologs of a triad are significantly different (defined by a PSI range of 20–80), and splicing bias is moderate; and (iii) Switched, in which two of the three PSI values for the homoeologs of a triad are extreme (with a PSI range of 80–100), and splicing bias is high. Triads in the balanced category contain homoeologs with conserved AS events and a conserved ratio of transcript abundance in each subgenome. Conversely, triads in the unbalanced category contain homoeologs with a biased ratio of transcript abundance among three subgenomes, indicating homoeologous divergence at the transcript level. Triads in the switched category typically contain one homoeolog encoding one major transcript (e.g. PSI < 10), and another homoeolog encoding a different major transcript in the same AS event (e.g. PSI > 90), indicating homoeologous transcripts that evolved independently through variation in AS into different dominant gene isoforms for their respective subgenomes. The distribution of AS events across the range of PSI values was bimodal across the three triad categories (Figure 5a), with clear separation of the categories in ternary diagrams based on PSI values (Figure [Link], [Link]A). During grain development, dynamic changes in the distribution of the three categories of splicing bias were observed (Figure 5b). In contrast to the late‐stage embryo, the early‐stage embryo contained a small proportion of AS events in the biased category and a greater proportion of AS events in the switched category (tested by DMRT), indicating AS bias is favoured in different stages of grain development, and variation in the AS regulation of wheat subgenome homoeologous transcripts is prevalent.

Biased regulation among homoeologous genes can result from GE bias or AS bias. GE bias in polyploid wheat has previously been defined (Ramírez‐González *et al.,* 2018; Xiang *et al.,* 2019). To define AS bias, GE and PSI bias were calculated for each triad (according to the GE bias criteria defined by Ramírez‐González *et al.,* 2018). One balanced and six homoeolog‐dominant or homoeolog‐suppressed groups were defined, including A, B and D subgenome‐dominant and A, B and D subgenome‐suppressed groups. Consistent with previous studies in wheat, most AS events and genes were assigned to the balanced group (82.1%–84.2%) (Xiang *et al.,* 2019; Yu *et al.,* 2020). Moreover, this analysis revealed a higher proportion of homoeolog‐suppressed genes (with 11% in subgenome A, 13% in subgenome B, and 12% in subgenome D) than homoeolog‐dominant genes (with 4% A, 3% B and 2% D subgenome‐dominant genes). Conversely, a higher proportion of homoeolog‐dominant AS events (11% A, 10% B and 8% D subgenome‐dominant AS events) than homoeolog‐suppressed AS events (6% A, 7% B and 6% D subgenome‐suppressed AS events) were identified. The opposing distribution of GE bias and AS bias identified among homoeologous genes indicates the divergent mechanism involved in the regulation of genes and isoforms.

To further discriminate between GE and AS biased regulation, we identified a set of triads with balanced GE and biased splicing, which included triads in the unbalanced and switched categories. This set included 2337 triads exhibiting balanced GE with biased AS across the seven grain developmental stages and tissues (Figure 5c,d, Data [Link], [Link]). Among these triads, a greater proportion of biased AS events fit into homoeolog‐dominant groups than into homoeolog‐suppressed groups, and more biased AS events were derived from the A and B subgenomes than from the D subgenome (Figure [Link], [Link]B). With balanced GE, the distribution of biased AS in developing grain tissues showcased elevated events in late‐stage embryo development, and the prevalence of AS events in the embryo, over the endosperm and pericarp (Figure [Link], [Link]C). The distribution of biased AS occurrences across embryogenesis, based on appearance ratios with balanced GE, identified 23 biased AS events that were consistent across all seven stages of embryo development (Figure [Link], [Link]D, Data [Link], [Link]). For the set of 23 conserved biased AS events, functional analysis of the corresponding genes showed enrichment of transcription factors (MYB, GATA, NFBY and AP2) and development‐related genes (e.g. auxin‐related), supporting key functions for splicing regulation during embryogenesis in wheat. In addition, splicing‐related genes were identified in the biased AS event set, encoding RNA binding proteins, RNA recognition motif‐containing family protein and arginine/serine‐rich splicing factors, which may belong to the core modulator set of genes that regulate splicing in polyploid wheat and their diploid ancestors.

Dynamic changes in gene expression patterns during embryogenesis, from stage E01 to E07, entail differential regulation. To add to the genetic complexity of embryogenesis, deviations in AS overdevelopment can yield transcript abundance differences, even in the absence of detectable gene expression differences. Therefore, identifying differences in splicing among subgenome homoeologs is critical in our efforts to tease apart the genetic underpinnings of grain production in polyploid wheat species. Although gene expression patterns among the three subgenomes' homoeologs in hexaploid wheat have been assessed (Xiang *et al.,* 2019), identifying AS events that alter transcript abundance, where expression differences were not previously identified, offers an opportunity to identify novel regulatory changes that impact embryogenesis and grain formation in polyploid wheats. To investigate the prevalence of transcript abundance changes due to AS, we performed pairwise correlation analysis among the three members of AS triads based on PSI and GE values of corresponding genes, to identify conservation of gene expression among homoeologs with differential transcript isoform abundance. Of the 1950 conserved AS triads, 186 had conserved patterns of GE and differential transcript isoform abundance during embryogenesis (Data [Link], [Link]). From the 186 conserved AS events, two AS events, Event 0691, associated with a gene encoding a WD40 repeat‐containing domain protein, and Event 0617, associated with a gene encoding a dehydration responsive element‐binding factor protein were selected at random, and their AS and GE patterns were compared in ternary diagrams to monitor GE and AS dynamics in the three subgenomes (Figure 5e–f, Figure [Link], [Link]A,B). Events 0691 and 0617 exhibited conserved homoeolog gene expression during embryogenesis (Figure 5e, Figure [Link], [Link]A), and divergent AS (Figure 5f, Figure [Link], [Link]B). Plots of GE trends using TPM and AS trends using PSI for the representative AS events were remarkably divergent, with consistent GE trends in the three homoeologs (Figure [Link], [Link]C,E), and variable AS trends in the three corresponding event triads during embryogenesis and grain development (Figure [Link], [Link]D,F). Thus, in addition to the dynamic patterns of GE over embryogenesis in wheat, AS variation among subgenome homoeologs can alter transcript abundance, providing an additional avenue for genetically regulating embryogenesis and grain formation in polyploid wheats.

### An evolutionary perspective on AS triads

**Figure 6:**
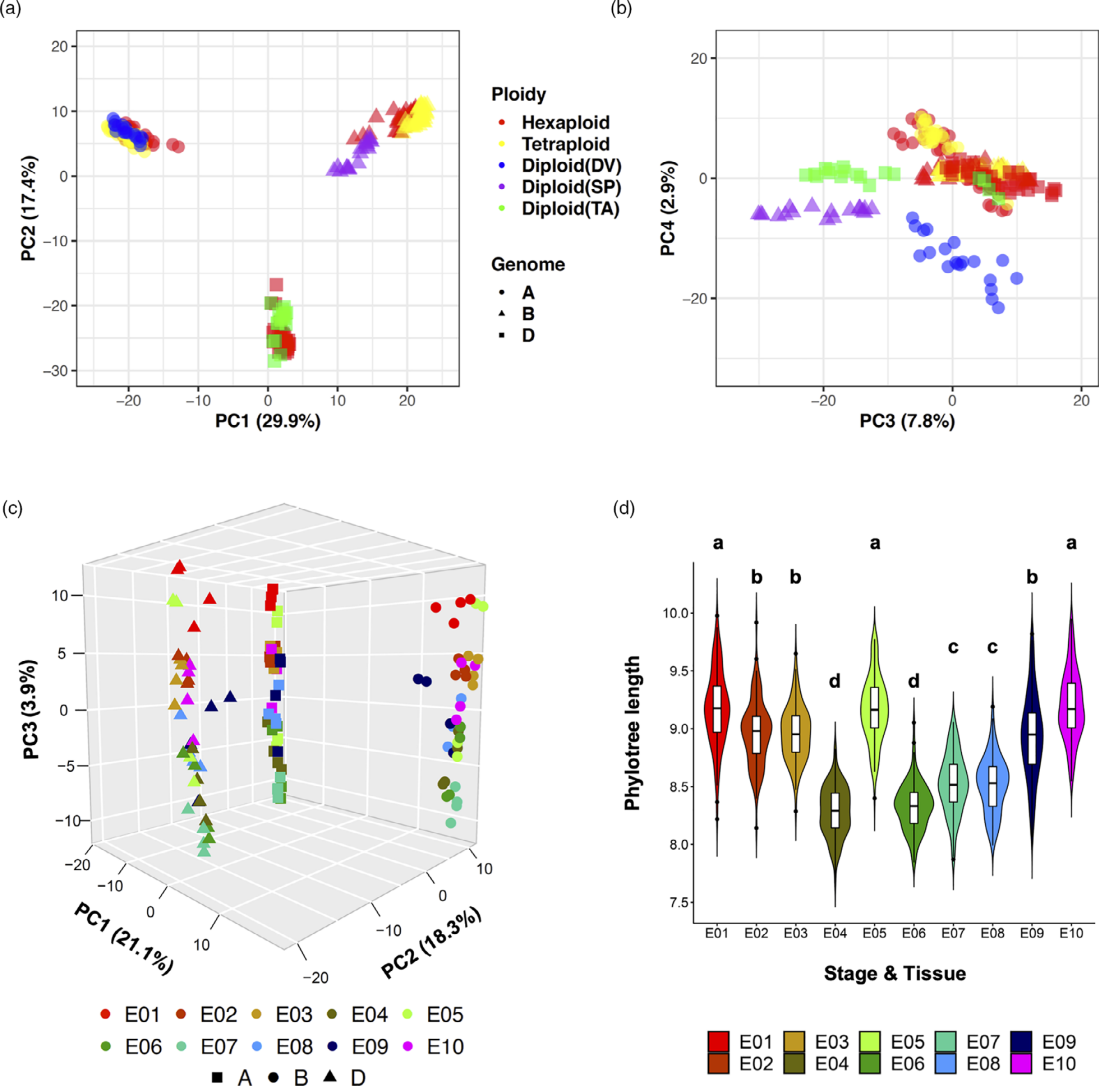
Comparative profiling of AS in related wheat and grass species by Principal Component Analysis (PCA) of conserved AS triads. (a) PCA based on PSI values from conserved AS triads in hexaploid and tetraploid wheats, and grass diploids DV, SP and TA, at the subgenome level. *X*‐axis and *Y*‐axis indicate two principal components (PC), PC1 and PC2, respectively. Ploidies and species are colour‐coded, and subgenomes are labelled with different shapes, as detailed in the inset. (b) PCA as in (a), in which *X*‐axis and *Y*‐axis indicate PC3 and PC4, respectively. (C) PCA 3D plot, based on PSI values from conserved AS triads in hexaploid wheat (for the AC and CS varieties) at the subgenome level. The fraction of variance explained by each principal component is indicated in the following bracket. Different stages and tissues are colour‐coded and subgenomes are labelled with different shapes, as detailed in the inset. (d) Violin plot for comparisons of total phylotree lengths (bootstrapping, 100 replicates) of expression trees from analysing three subgenomes of hexaploidy wheat (AC, CS) using conserved AS triads. Statistically significant differences among different stages/tissues are indicated on the top of each violin (Duncan's tests). Full expression trees for each stage/tissue are shown in Figure [Link], [Link].

Our PCA analysis of PanAS events identified ploidy as the primary determinant of AS pattern variability, and developmental stage and grain tissue as secondary determinants. After identifying above homoeologous AS triads, subgenome factors could be included in evolutionary analyses. Using the 1,950 conserved AS triads from the five species across grain development, a 2‐D PCA analysis based on PSI values was performed to compare the contributions of subgenome, ploidy, stage and tissue to the AS pattern variability (Figure 6a,b). The first and secondary components (PC1 and PC2) separated all samples by subgenome, contributing 50% explanation of variance (Figure 6a, Figure [Link], [Link]A), followed by ploidy in PC3 and PC4, which explained approximately 10% of the variance (Figure 6b, Figure [Link], [Link]A). Similar PCA results were obtained when the influence of ploidy to sample variance was excluded (Figure 6c). Using the two hexaploid wheat cultivars, AC and CS, a PCA 3‐D plot showed clear segregation of subgenomes in PC1 and PC2, contributing approximately 40% explanation of variance, while PC3 suggested stage and tissue differences contribute 3.9% explanation of total variance. Unsupervised PCA clustering results were validated by PLS‐DA analysis using the same AS triad sets and forcing separation by (i) subgenome and (ii) ploidy (Figure [Link], [Link]). For both the subgenome‐forced PLS‐DA and the ploidy‐forced model, clustering of samples by subgenome was observed (Figure [Link], [Link]A,B). These clustering results reveal the strength of subgenome differences for explaining AS variation in polyploid wheats and their diploid ancestors. From our gene isoform level clustering analyses, the evolutionary divergence of transcript regulation due to AS for polyploid wheats and their putative ancestors appears to be influenced by, in descending order, the subgenome, ploidy level and developmental stage/grain tissue. This trend is distinct from clustering analyses based on gene expression differences, in which the developmental stage and tissue of wheats and diploid grass grains contributed the largest variance (Xiang *et al.,* 2019), supporting divergent gene‐level and transcript‐level regulation of grain development in polyploid wheats and their diploid ancestors.

With data supporting the emergence of divergent splicing events in the three subgenomes of hexaploid wheat, we next examined how this divergence manifests in the subcompartments of wheat grains overdevelopment. Using the two hexaploid wheat cultivars, AC and CS, phylogenetic trees of the 1950 AS events in the three subgenomes were generated for each stage of embryogenesis (Figure [Link], [Link]). The sum of phylogenetic tree branch lengths for each stage/tissue was used to estimate evolutionary divergence between the AC and CS cultivars. With the exception of E05, longer tree branch lengths were detected in early stages of embryo development than in late stages, indicating more divergent evolution in the initial establishment and patterning stages of embryogenesis (Figure 6d). AS detected in the embryo displayed less divergence than the endosperm and pericarp in the same stages, supporting stronger conservation in the regulation of splicing in the embryo than in other grain tissues (Figure 6d, Figure [Link], [Link]).

### Evolutionary age of transcript isoforms during embryogenesis in wheat polyploidization

**Figure 7:**
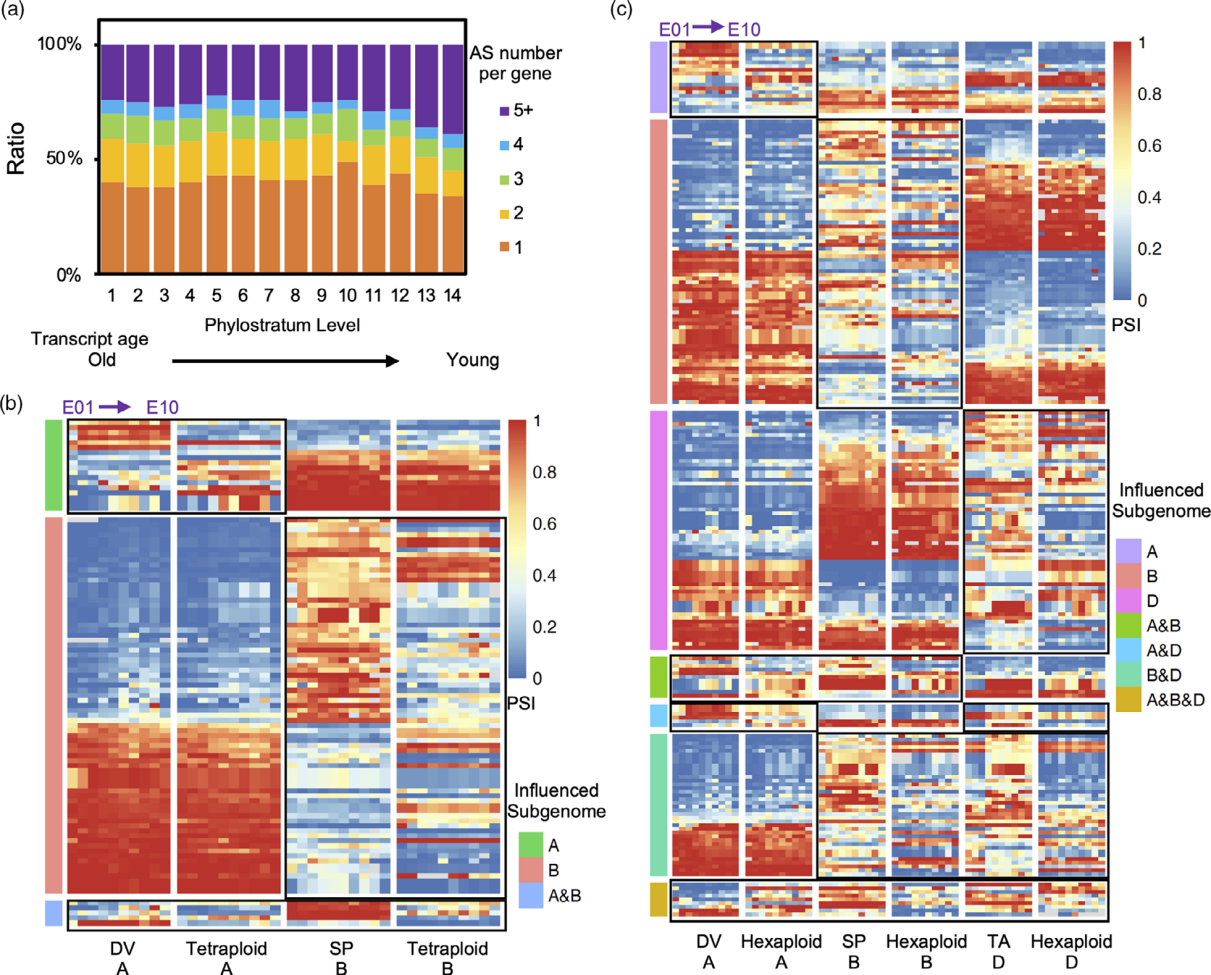
Analysis of transcript age and the effects of polyploidization in polyploid wheat (a) Distribution of the AS events per gene in each of the 14 phylostratum (PS) levels. *X*‐axis, PS levels, from left to right, PS1 (oldest genes) to PS14 (youngest genes). *Y*‐axis, stacked gene ratio displaying the proportion of AS events, from 1 to 5+, as indicated by the right inset colour scale. (b–c) Heat maps of conserved AS event triads, in which PSI values appear significantly influenced by polyploidization in (b) tetraploid and (c) hexaploid wheat. Sequential colour scales in the heat maps indicate PSI values, from blue (0) through yellow (0.5) to red (1). Different categories of AS triads are clustered based on the influence of subgenomes. These categories are grouped by colours to the left of heat maps (as defined in the inset), and influenced subgenomes in each category are outlined with black rectangles. Different stages/tissues from E01 to E10 in the same subgenome sequentially arranged from left (E01) to right (E10) in each cluster. Subgenomes are indicated along the bottom of each heat map.

Evolutionary age and sequence divergence have been used to predict transcriptome age (Domazet‐Lošo and Tautz, 2010; Quint *et al.,* 2012). To assess the evolutionary age of transcript isoforms during grain development in polyploid wheats and their putative ancestors, 14 phylostratum (PS) levels, including Cellular Organism, Eukaryota, Viridiplantae, Embryophyta, Tracheophyta, Magnoliophyta, Mesangiospermae, Commelinids, Poaceae, BOP clade, Pooideae, Triticeae, Triticinae and Triticum were identified based on a phylostratigraphic map (as detailed previously by Xiang *et al.,* 2019). Each isoform was assigned to one PS level from PS1 (representing the most ancient isoform) to PS14 (representing the youngest isoform). As shown in Data S13, identified AS events (5 < PSI < 95 and TPM > 1) were selected for evolutionary age prediction, and the distribution of total AS events from each gene was analysed for PS levels (Figure 7a). Notably, younger genes (belonging to PS13 or PS14) exhibited significantly higher ratios of expressed transcript isoforms (>5) per gene than older genes (tested by DMRT), indicating that the AS in young genes generally appears to have evolved at a more complex pattern than in old genes, by forming novel transcript isoforms.

Polyploidization is a major driving force for diversification, providing the opportunity for accelerated genome modification with the potential for novel gene functions and trait innovations in plants. Studies to understand the gene expression of subgenome homoeologs and how such genome modifications are linked to grain traits in polyploid wheats and their diploid ancestors provide insight into the role genome duplication has played in the evolution of modern wheat crops (Ramírez‐González *et al.,* 2018; Xiang *et al.,* 2019). To investigate evolutionary divergence after polyploidization, we identified the AS events with significantly different PSI in polyploid wheats in comparison with their diploid ancestors (Figure 7b,c, Data S14). Between any of the two diploid species, differences in PSI values greater than 50 (PSI_diploid ancestor 1_ − PSI_dipoloid ancestor 2_ > 50) in a conserved AS triad were selected as a cut‐off to identify triads in which dominant transcript isoforms differed among diploid species. The evolutionary trajectory of these AS triads or diads was tracked in polyploid species using PSI values. For the tetraploid and hexaploid wheat species in this study, 98 AS diads and 223 AS triads were, respectively, identified as significantly different in comparison with their diploid ancestors (PSI difference > 25, *P*‐value < 0.05) (Figure 7b,c). 18 AS diads showed similar PSI values in the B subgenome of the CM and ST tetraploid wheat cultivars, as their putative diploid ancestor SP, but had significantly altered PSI values in the A subgenome in comparison with the diploid DV. 75 AS diads exhibited relatively constant PSI values in the A subgenome of CM and ST when compared to the DV putative diploid ancestor, with significantly altered PSI values in the B subgenome of these tetraploid wheats, in comparison with SP. The tetraploid species contained five AS diads with altered PSI values in both A and B subgenomes compared to their diploid ancestors during grain development (Figure 7b, Data S14). In hexaploid wheat species, comparison of two cultivars (CS and AC) to the three putative ancestral diploid grasses shows that the evolutionary trajectory of AS triads was complex, with single‐, dual‐ and triple‐subgenome alterations (Figure 7c). Specifically, 19, 76 and 64 AS triads exhibited single‐subgenome PSI alteration in the A, B and D subgenomes, respectively. For dual‐subgenome PSI alterations influencing the A&B, A&D and B&D subgenomes, 11, 6 and 38 AS triads were identified, respectively. Lastly, 9 AS triads were significantly altered in all three subgenomes in comparison with their putative diploid ancestors (Figure 7c, Data S14). Together, the relatively fewer AS events in the A subgenome compared to the B and D subgenomes indicates higher conservation of gene expression regulation through AS in the A subgenome during grain evolution in polyploid wheats. Furthermore, we found 9 (from A subgenome) and 36 (from B subgenome) AS triads with PSI change in both tetraploid and hexaploid species, in comparison with the diploid ancestors. These triads contain several transcription factors, including BHLH and MYB‐types, which regulate the seed coat differentiation and seed germination (Feller *et al.,* 2011), as well as storage reserve‐related genes, such as alpha/beta hydrolase. Uncovering the mechanisms of differential splicing regulation in these triads and their impact on grain traits in diploid vs. polyploid wheat species will provide valuable insight on the impact of domestication on grain traits. Three AS triads showed altered PSI values in all subgenomes of tetraploid and hexaploid species compared to their diploid ancestors. The corresponding genes of these three AS events encode F‐box proteins, ubiquitin‐conjugating enzyme E2 and protein‐tyrosine phosphatases, indicating some protein level modifications in the embryo were reformed after polyploidization in wheat.

### Validation of AS events through droplet digital PCR (ddPCR)

**Figure 8:**
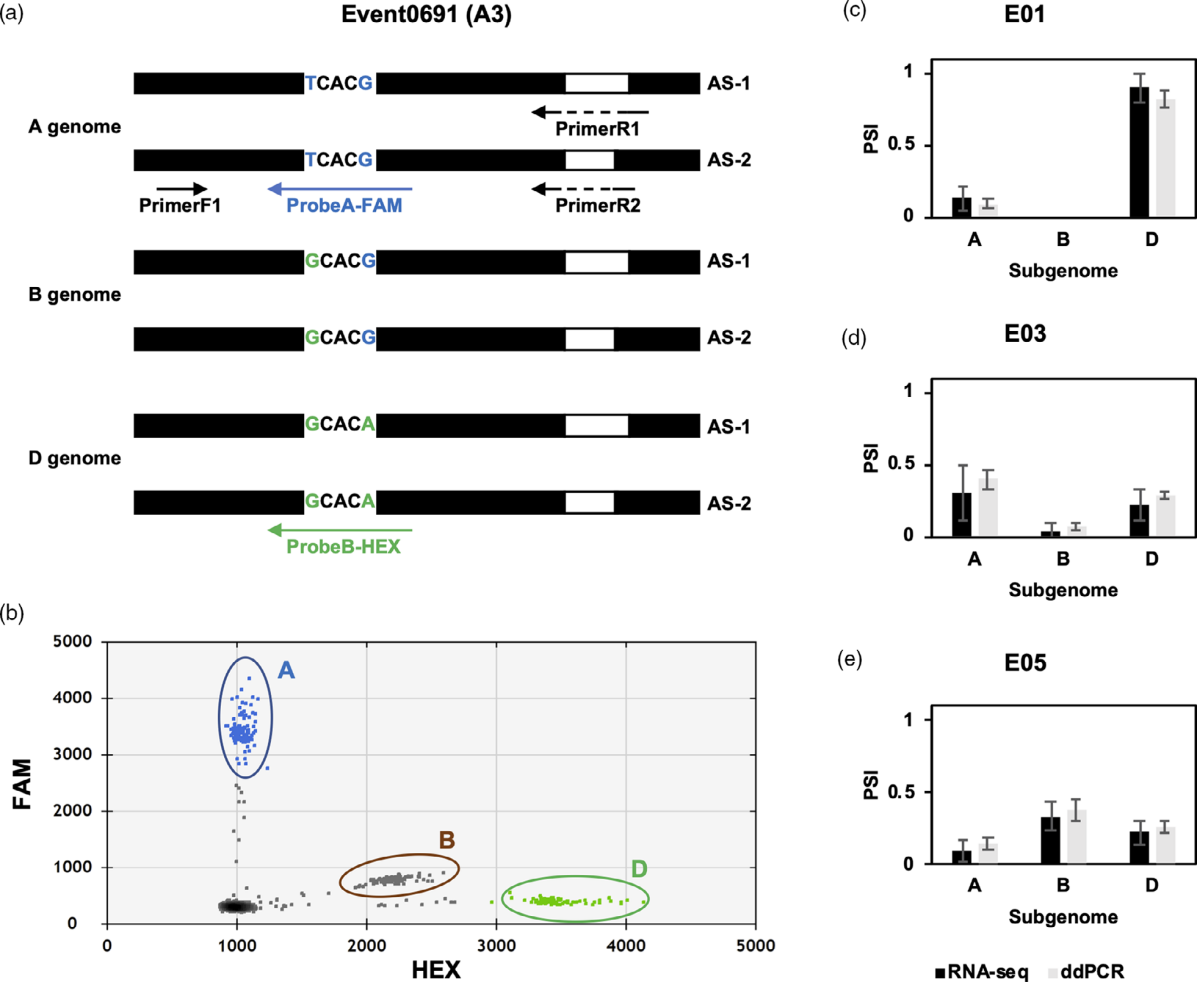
Validation of AS triads by droplet digital PCR (ddPCR). (a) Example of validation strategy applied, for an A3 type AS triad – Event0691. Structures of PCR product regions containing two different AS junctions (AS‐1 and AS‐2) in three subgenomes are shown. Splicing junctions span reverse primer R1 or R2. Two SNPs (T‐G and G‐A) across three subgenomes are labelled in blue and green, which were further included in two fluorescent probes (FAM and HEX). Black box, exon; white box, intron. (b) 2‐D droplet amplitude plot for AS‐1 transcript of Event0691 in three subgenomes of AC Barrie in the E03 stage. Clusters of droplets representing three subgenomes were surrounded by ovals with three different colours. *X*‐axis, the amplitude of droplets in HEX channel; *Y*‐axis, the amplitude of droplets in FAM channel. (c–e) Comparison of PSI from RNA‐seq analysis using our pipeline and ddPCR analysis. (c–d) AC Barrie embryo samples from stage E01 (c), E03 (d) and E05 (e) were used for comparative analysis. No significant difference between PSI values from RNA‐seq analysis and ddPCR validation were detected, for all three subgenomes (A, B and D). Black columns, PSI from RNA‐seq analysis; grey columns, PSI from ddPCR analysis using our custom method.

In wheat, the high sequence similarity of homoeologous genes makes it difficult for molecular validation of AS. To address this problem, we developed a cost‐effective method to detect all three homoeologous genes and transcript isoforms in one ddPCR reaction, significantly decreasing the cost of AS validation. The design of AS site‐spanning primers and two fluorescent probes with 2–3 base pair mismatches (Figure 8a, detailed in Methods), enabled accurate detection and quantification of transcript isoforms from each homoeologous gene (Figure 8b). Using this method, we examined the expression level of the dynamic Event0691 AS triad in the A, B and D subgenomes of wheat. Transcripts from each subgenome in hexaploid CS were detected across the seven grain developmental stages (Figure 8c–e). A high correlation of transcript ratio (PSI) was found between the genomic results of this study and the results of ddPCR analyses (Pearson's correlation coefficient *r* = 0.89), thus supporting the conclusions of RNA‐seq based global data sets and our downstream analyses.

## Discussion

The expansion of plant genomes by polyploidization provides a platform for novel patterns of gene expression and transcript processing, and thus, the opportunity to accumulate novel traits over evolutionary time. Uncovering regulatory GE and transcript splicing changes, including variation between subgenomes, in agriculturally valued polyploid crops such as hexaploid *T. aestivum* and tetraploid *T. turgidum* ssp. *durum*, will enable the impact of genetic changes on the evolution of the grain to be deciphered. Further, insights on the regulation of GE and AS in polyploid wheats will inform efforts to alter seed/grain composition traits. Here, we developed a custom bioinformatics pipeline and a cost‐efficient validation method through ddPCR to quantify transcript isoforms and identify AS events in five related ancestral grass diploid and polyploid wheat progenitor species with high levels of sequence similarity. By applying this platform for AS analysis to transcripts in the major tissues of the grain, throughout embryogenesis, we were able to make comparisons among subgenome homoeologs, and identify differences in gene‐level and transcript‐level regulation. Through our analysis of GO term enrichment in the PanAS category, remarkable differences in AS regulation were identified in the photorespiration category, suggesting substantial transcript‐level processing differences may impact photorespiratory pathway functions in the developing grain among polyploid wheat and diploid grass ancestors. Finally, through our development of a novel bioinformatics pipeline, this study produced an extensive list of specific AS events in the subcompartments of the developing wheat/grass grain, including the developmental distinction of early‐ and late‐stage embryos, endosperm and pericarp. Transcript‐level variations in genes related to key grain developmental processes, such as in storage and starch accumulation, offer opportunities to target grain traits for crop optimization.

### A new pipeline optimized for AS analysis in polyploid species

Although several methods for transcript‐level expression analysis have been published, for example the HISAT‐StringTie‐Ballgown pipeline (Pertea *et al.,* 2016), and some have been applied to the study of polyploid plant species, optimization of classic bioinformatic pipelines to address the high sequence similarity issue in polyploid plants for AS analysis was lacking. In this study, we created a customized bioinformatics scheme to quantify transcript isoforms and identify AS events in the subcompartments of developing grains of polyploid wheats and their putative ancestors, thereby improving the accuracy of AS event detection in polyploid species (see pipeline section in Methods). In comparison with the HISAT‐StringTie‐Ballgown pipeline, our optimized workflow used STAR alignment, applying the manual 2nd pass mode protocol for multiple samples to collect high confidence splicing junction information. StringTie was used for transcript assembly and transcript merging, and quantification of transcripts was performed through realignment using Salmon, to distinguish highly similar homoeologous sequences in polyploid species. After comparing several AS detection tools (Ding *et al.,* 2017), tools utilizing paired biological replicates (e.g. SUPPA2 and rMATS) were chosen to diminish individual variation and increase statistical power. AS event identification and differential splicing analysis by SUPPA2 and rMATS provided detailed classification and accurate PSI values, which substitute the Ballgown tool for differentially expressed transcript analysis. For molecular validation of splicing junction and PSI values in polyploid plants, we optimized a ddPCR method for polyploid species, offering a cost‐efficient tool for high‐throughput validation of RNA‐seq and downstream analysis processes. Combined with further analysis of specific AS events and conserved homoeologous AS events, the pipeline detailed in this study provides an instructive workflow for evolutionary analysis of AS in polyploid species at the transcript level.

### RI and A3 types of AS during grain development in wheat species

Although RI is not an abundant AS event in humans (accounting for 9% of AS events) (Kim *et al.,* 2008), approximately 40% of AS events in *Arabidopsis* produce RI isoforms (Ner‐Gaon *et al.,* 2004). In select monocots, including maize and sorghum, RI has been identified as a major AS event type (Wang *et al.,* 2018a), and a slightly higher proportion of A3 over RI was found in maize and rice (Barbazuk *et al.,* 2008). In this study, we detected a higher proportion of A3 type AS events during embryogenesis in wheat, followed by relatively fewer RI events, in comparison with other plant species (Figure [Link], [Link]). There are several possible reasons for this observed RI proportion difference. First, our profiling of AS events by type used SUPPA2, a tool that is sensitive to A3 and A5 events but less sensitive to RI events, especially for double or multiple intron retentions. Although RI event counts may be reduced, this approach facilitated an increased detection of other types of AS events, supplemented by ddPCR methods for validation (Figure 8). The compatibility of SUPPA2 with the Salmon tool in our pipeline was also important for detecting homoeologous transcripts and reducing the interference of genomic DNA contamination (an unexpected source of false‐positive RI events) during sequencing. Second, 10 < PSI < 90 is typically used as the filtration threshold to select high confidence AS events. Lowering this threshold (to 5 < PSI < 95) for increased sensitivity enabled reliable AS events from our high coverage of sequencing depth to be selected. In this way, we did identify some AS events with extremely high or low PSI, which are validated by our modified ddPCR method (Figure 8). On the other hand, we found raising the filtration threshold to 10 < PSI < 90 for our data set increased the proportion of RI by approximately 10% and decreased the proportion of detected A3 and A5 AS events, suggesting that those AS events with extremely high and low PSI values mainly belong to the A3 or A5 categories. Lastly, transcript splicing events have been shown to vary in different plant tissues, and consistent with our findings, a lower proportion of RI splicing events in the embryo and endosperm tissues of the seed has been reported in monocot species (Thatcher *et al.,* 2016; Wang *et al.,* 2018a). In support of this, in human, it has been found that the percentage of the most frequent AS event (exon skipping, ES) varies considerably from 10% in the ovary to over 30% in the brain, while A3 and A5 events vary considerably from about 4% to close to 30% depending on different tissue types (Yeo *et al.,* 2004). The similar regulating mechanism for AS type distribution probably exists in plants and could contribute to the reduced proportion of RI detected herein.

To examine and validate our AS event profiling results, we applied another AS detection method, using the HISAT‐StringTie‐Ballgown pipeline in combination with ASTALAVISTA (Foissac and Sammeth, 2007), a web server that can extract AS events, to detect AS events from diploid species samples. By selecting diploid species to avoid the influence caused by the similar homoeologous transcripts in polyploid species, this method still did not identify RI as a major type of AS in our samples, with 22% in DV, 23% in SP and 25% in TA were identified as the RI event type. These results suggest that the mechanisms of AS site recognition and/or bias for certain AS event types between plant species and amongst tissues could promote different fates for the embryo and endosperm during grain development. The origin and biological significance of the reduced proportion of RI detected in this study warrants further investigation.

Although our analyses did not support RI as a prominent feature in embryogenesis of wheat species, RI appeared to play an important role during gene isoform evolution. In contrast to the four other major junction splicing types, RI was the only type of AS that exhibited significant variation in abundance (Figure 1c,d). In comparisons between all AS events and select categories of AS, such as PanAS and VariantAS, the frequency of RI was elevated relative to the other four major junction splicing types (Figure 2a). This indicates a bias towards RI for splice site recognition of uncommon AS events in all stages/tissues and species, revealing a key regulatory role for RI events in wheat species during grain development.

### AS specificity across stages, tissues and species

Through our evolutionarily rooted comparative analysis of transcript‐level regulation in wheats and ancestral grasses, an array of AS events was detected in the tissue subcompartments of developing grains for different wheat and diploid grass species. Many splicing events were specific to a particular stage or tissue and thus may be regulated in a stage‐ or tissue‐specific manner. The expression of different AS isoforms across the tissues of a grain can produce gain or loss of functional domains and cause tissue‐specific functionality changes (Modrek and Lee, 2003). For example, in the middle embryonic stage of development, the endosperm possessed more tissue‐specific AS events than the embryo and pericarp (Figure 4f,g). The up‐regulation of gene expression relating to the storage of starch and storage protein in polyploid wheat embryogenesis was previously shown to be initiated at the middle embryonic developmental stage, and enriched in the endosperm (Xiang *et al.,* 2019). Similarly, our data suggest AS regulation may impact starch and storage protein accumulation processes and the associated grain quality traits through the endosperm‐specific AS events at middle embryonic developmental stage (Figure 4f,g). Similar to the tissue‐specific gene expression analysis, E01 and E07 showed AS event enrichment relative to the other stages of embryogenesis. Intriguingly, there was almost no overlap in the enrichment of GO terms from the genes associated with stage‐specific AS events and the stage‐specific expressed genes (Figure 4g). The lack of overlap between the splicing and gene expression suggests that for complex biological processes, an isoform splicing regulatory mechanism exists to complement gene expression regulation.

AS events specific to the three subgenomes of diploid, tetraploid and hexaploid wheat were identified. A higher number of AS events were identified in the diploid species examined, especially in SP and TA (Figure 4a–e), suggesting some AS events are not retained after polyploidization, and the biological function of absent isoforms may be replaced by the homoeologous transcripts of other subgenomes. We investigated altered AS events after polyploidization using AS triads and identified several groups of AS triads in different subgenomes (Figure 7b,c). Among these specific and altered AS events, a number of genes and transcription factors involved in seed germination, dormancy and seed trait related processes (Figure 4 and Data S8), which is consistent with previous AS data for the leaf and roots of hexaploid wheat (Yu *et al.,* 2020). Functional analysis of these AS events might shed light on the evolutionary process of how polyploid wheats were adapted and domesticated after polyploidization.

### Evolutionary divergence in AS and GE regulation

Studies in animal species have revealed different evolutionary rates of regulation at the gene and transcript levels, after the separation of alternatively spliced exons among species ~6 million years ago (Barbosa‐Morais *et al.,* 2012; Calarco *et al.,* 2007). In our study, PCA analyses identified different rates of evolution of AS and GE in polyploid wheat during grain development (Figure 3 and Figure [Link], [Link]). When clustering AS profiles, the samples from seven polyploid wheats and their putative ancestors were separated by ploidy, which is in contrast to clustering of GE profiles by stage and tissue. As discovered in animal species, the major source of AS diversity is explained by the species, while the major source of GE diversity arose at the tissue‐level (Barbosa‐Morais *et al.,* 2012). These differences in the regulation of AS and GE reflect divergent evolutionary processes of gene expression and AS level regulation in plants. A recent study investigating the regulation of AS and GE in Arabidopsis under stress conditions (Martín *et al.,* 2021) found the core genes regulated by AS and GE in response to internal and external stimuli shared few common members. One clear difference is that ﻿RNA binding proteins (RBPs) were significantly enriched in the AS core sets, whereas GE core gene sets were depleted in RBPs but significantly enriched in DNA‐binding proteins. Similarly, in this study, a batch of RNA binding proteins and RNA recognition motif‐containing family proteins enriched in identified AS event sets, including tissue‐, stage‐, species‐specific, biased and polyploidization‐altered AS events, indicating this regulatory specialization is widespread. Moreover, this AS landscapes study in Arabidopsis discovered that the different AS event types were differentially regulated under stress or in different tissues with RI showing the most diverse changes (Martín *et al.,* 2021). This is similar to our conclusion on the comparison of AS event types among developmental stages, tissues and subgenomes (Figure 1), revealing the complexity of AS spatiotemporal regulation as a key element in gene expression.

Further investigation of AS triads suggested that subgenome differences explained many of the transcript‐level differences among polyploid wheats and their putative ancestors (Figure 6a), by holding numerous Switched and Unbalanced AS triads among three subgenomes (Figure 5a). Some gene triads showed a similar gene expression trend among the three homoeologous members, but had completely distinct transcript expression trends during embryogenesis (Figure 5e,f and Figure [Link], [Link]), indicating that AS might play a complementary role for complex processes of gene regulation. As shown in Figure 7a, young genes developed more isoforms per gene loci compared to ancient genes, suggesting this complementary mechanism of transcript‐level regulation was accelerated under the selective pressures of domestication in polyploid wheats.

## Materials and Methods

### Plant material, growth and isolation of embryo, endosperm and pericarp

Tetraploid wheat (AABB, *T. durum*: CDC Commander‐CM) and hexaploid wheat (AABBDD, *T. aestivum*: Chinese Spring‐CS) were used to perform further deep‐sequencing of samples collected from sequential developing stages of grain development (defined as E01–E10). In addition to the tetraploid and hexaploid wheats selected here, Xiang *et al.,* 2019 previously isolated these key subcompartments of the developing grain for five related species of polyploid wheats and their diploid ancestral species, including three diploids *T. monococcum* (AA, DV92‐DV), *Ae. speltoides* (BB, TA2780‐SP) and *Ae. tauschii* (DD, TA101132‐TA), tetraploid wheat (AABB, CDC Strong field‐ST) and hexaploid wheat (AABBDD, AC Barrie‐AC), as summarized in Table [Link], [Link]. Here and in Xiang *et al.,* 2019, the tissues and developmental stages examined included the embryo at seven stages, from the zygote to maturity (two cell embryo – E01, pre‐embryo – E02, transition – E03, leaf early – E04, leaf middle – E05, leaf late – E05 and mature embryo – E07), the endosperm in two stages (the transition stage endosperm – E08 and leaf late‐stage endosperm – E09), and the pericarp in one stage (leaf early‐stage pericarp – E10). Polyploid (AC, CS, CM and ST) wheat plants and one diploid grass (DV) were grown in growth chambers under long‐day conditions of 16 h light, 22 °C and 8 h dark, 20 °C and 100–120 μmol m^−2^ s^−1^ light intensity for the entire life cycle. The other two diploid grasses (SP and TA) were initially grown in a growth chamber at 22 °C under long‐day (16 h day/8 h night) conditions. At the fifth‐leaf stage, vernalization treatment was carried out at 4 °C for 1 month under a long‐day photoperiod; then returned to 16 h light, 22 °C and 8 h dark, 20 °C; light intensity: 100–120 μmol m^−2^ s^−1^ growth conditions to complete the life cycle. Embryo, endosperm and pericarp tissue isolation procedures were performed precisely as described previously (Xiang *et al.,* 2019). To meet the requirement of downstream RNA‐seq, sufficient sample materials from each of the three grain subcompartments (especially in early‐stage embryos) were isolated and washed.

### Microscopy

For scanning electron microscopy (SEM), a longitudinally cut AC Barrie grain was submerged in 25 mm PIPES, pH 7.0 containing 2% v/v glutaraldehyde for 2 h at room temperature. After several washes, samples were fixed in 2% OsO_4_ in 25 mm PIPES for 2 h, washed in 25 mm PIPES and dehydrated in an ascending ethanol gradient (30%, 50%, 70%, 95% and three 100% exchanges). After sample dehydration, substitution to amyl acetate was performed with increasing ratios of amyl acetate to ethanol (1:3, 1:1, 3:1, then two pure amyl acetate exchanges). All solvent exchanges were separated by 15 min. Samples were critical‐point dried with solvent‐substituted liquid CO_2_ (Polaron E3000 Series II, Quorum Technologies, Lewes, UK), mounted on aluminium specimen stubs with conductive carbon glue (Ted Pella, Inc., Redding, CA, USA) and rotary coated with 10 nm of gold (Edwards S150B sputter coater, BOC Edwards, Crawley, UK). Imaging was performed with a 3 kV accelerating voltage, 10 uA current and 12.2 mm working distance on a Field Emission SEM (Hitachi SU8010, Hitachi, Tokyo, Japan).

### RNA isolation, aRNA amplification and RNA‐seq analysis

Total RNA was extracted from embryo, endosperm and pericarp tissues at different developmental stages for each wheat or grass species (AC, CS, CM, ST, DV, SP and TA) following the RNAqueous‐Micro kit protocol (Ambion AM1927, Thermo Fisher Scientific, San Jose, CA, USA, Catalog# 1927). Two biological replicates for each sample were collected. As the quantity of RNA isolated from early embryonic developmental stage was insufficient for library preparations of the RNA‐seq experiments, amplified antisense RNA (aRNA) was used for RNA‐seq analysis. aRNA generation and cDNA synthesis (used for validation) was conducted according to the protocol provided in the MessageAmp aRNA kit (Ambion, Catalog# 1750). For RNA‐seq profile analysis, Illumina mRNA‐seq libraries were constructed using the TruSeq RNA kit (version 1, rev A). To generate high coverage and accurate reads that meet AS analysis demands, Illumina HiSeq 2000 sequencing system was utilized and library numbers per sequencing lane were restricted to 3–4.

### Pipeline for splicing evolution analysis in polyploid plants

#### Alignment of RNA‐seq reads to reference genome

Obtained high coverage reads were filtered using the Trimmomatic tool (Bolger *et al.,* 2014) with the parameters set to ILLUMINACLIP:TruSeq3‐SE:2:30:10 SLIDINGWINDOW:5:20 MINLEN:75 to remove sequencing adapters and low‐quality reads. For all 140 samples (20 samples per species), the IWGSC RefSeq assembly v1.0 complete reference genome was used for preliminary mapping of trimmed reads, where corresponding subgenomes were used for diploid and tetraploid species. Cleaned RNA‐seq reads in each sample were mapped against the reference genome using the modified STAR (Dobin *et al.,* 2013) 2nd pass mode pipeline: First, preliminary mapping was based on the index generated from the reference genome mentioned above, and the argument ‐‐alignEndsType EndToEnd was included in the mapping process to avoid creating false junctions caused by default soft clipping. All junctions detected in the 1st pass can be collected from SJ.out.tab files in all runs, and these junctions were further filtered by removing false positives. For novel junctions which are not present in IWGSC RefSeq annotation v1.1, only reliable junctions supported by more than 5 reads were retained. Secondly, all SJ.out.tab files from the same species were merged and then used for generation of a new reference index for each species through the parameter ‐‐sjdbFileChrStartEnd. Finally, the 2nd pass mode of STAR alignment was performed again for all samples based on the new reference index. Generated BAM files were sorted for further transcript assembly.

#### Assembly of novel transcripts

Transcript assembly was performed using StringTie (Pertea *et al.,* 2015), based on the sorted BAM files. GTF files generated from each sample in the same species were merged together using IWGSC RefSeq annotation v1.1 as reference. Novel transcripts were then identified through Gffcompare (https://github.com/gpertea/gffcompare), and their corresponding genomic coordinates were transformed based on the reference genome, using a custom script. Transcript sequences in FASTA format were extracted using Gffread (https://github.com/gpertea/gffread) and the FASTA files generated for seven wheat cultivars and their ancestors including novel and known transcripts were used as transcript reference for further quantification.

#### Quantification of identified transcripts and genes

To address the issue of high sequence similarity of homoeologous genes and transcripts with AS, a quasi‐mapping tool Salmon (Patro *et al.,* 2017), which performed well in stringent homoeolog mapping based on the alignment of unique sequences of length k (kmer) in the transcriptome, was used for quantification of all transcripts identified in each species. Using Salmon with arguments ‐l A –validateMappings and newly generated FASTA transcripts reference, another fast and accurate alignment was performed for the cleaned reads. Salmon quantmerge function was then used to obtain transcripts per million reads (TPM) of each sample, which were then used for further PSI calculation. To aggregate expression levels from the transcript to the gene level (GE), we used R tximport package to process the TPM values obtained from Salmon. Transcripts with overlapping genomic coordinates were considered from the same gene, and expressed genes were determined based on the global expression of each gene across all stages/tissues in which it was expressed more than 1 TPM in at least one stage/tissue.

#### AS event profiling, filtering and PSI calculation

AS event type profiling was performed using SUPPA2 (Trincado *et al.,* 2018) and rMATS (Shen *et al.,* 2014) from the merged GTF annotation files obtained above, respectively. Seven event types can be generated by SUPPA2: A5, A3, RI, SE, MX, AF and AL; ioe format files were produced for each AS event in a gene describing either form of the event. Specifically, ioe files provide the transcripts that contribute to the numerator (one form of the event) and the denominator (both forms of the event) of the PSI calculation. The pool genes function of SUPPA2 was applied to cluster the overlapping transcripts that share a great deal of sequence and thus relative splicing events could be taken into account. PSI calculations were computed based on the TPM values and each event from the ioe file. Further filtering was conducted considering both *P*‐values (<0.05) from rMATS calculation and transcript expression. Events were filtered out if total expression of the transcripts in the event is less than 1 TPM (parameter −f 1). Filtered events and corresponding PSI values were combined for each species, and events with 5 < PSI < 95 in at least one stage/tissue were preserved for downstream analyses. PanAS was defined as AS events with 5 < PSI < 95 in at least 80% of stages/tissues across embryonic development. VariantAS was defined as AS events with PSI range >25 (maximum − minimum) across all samples. Bar plots and circular stacked bar plots for the number of different event types were plotted using R tidyverse and ggplot2 package (https://www.tidyverse.org/).

#### Identification of conserved AS in wheat cultivars and their putative diploid ancestors through chain file

To identify conserved AS triads across the three subgenomes of wheat, we established pairwise chain files between every two subgenomes for conserved exons identification. Chain files for the three wheat subgenomes were generated based on the modification of a workflow from UCSC Genome Browser (https://github.com/ENCODE‐DCC/kentUtils/blob/master/src/hg/utils/automation/doBlastzChainNet.pl). The lastz parameters used in this workflow were optimized specifically for the wheat genome and the final arguments adopted were BLASTZ_H = 2000, BLASTZ_M = 254, BLASTZ_O = 600, BLASTZ_E = 150, BLASTZ_K = 4500, BLASTZ_Y = 15 000 and BLASTZ_T = 2. The soft masked genome sequences of A, B and D subgenomes were applied, and three chain files with 1:1 orthology relationships (A to B, A to D and B to D) were generated. Conserved AS triads were selected using Lift‐Over tool (Haeussler *et al.,* 2019) through selecting 1:1:1 overlaps among three subgenomes using three chain files. Splicing bias among AS triads was calculated based on PSI and visualized by ternary diagrams using R ggtern package.

### Principal component analysis (PCA) of gene expression and transcript expression

Relative relatedness and reproducibility of genes or transcripts among all samples were examined by PCA using GE and PSI, respectively. For PCA of relatedness in transcript‐level for ploidy, stage and tissue in each subgenome, PSI of PanAS events from diploid, tetraploid and hexaploid wheat cultivars were used. Expression data matrices were comprised of logarithm transformed TPM values including the genes containing PanAS events, which were used for PCA of gene expression in each subgenome. To include the subgenomes in PCA, only conserved AS triads were adopted. For further comparison of GE between species, we calculated differential expression based on a method we established in our previous work, called differential expression feature extraction (DEFE) method (Pan *et al.,* 2019; Xiang *et al.,* 2019). This method is based on negative binomial distribution model and considering transcript length in the meantime, which is ideal for the comparison across species with different transcriptome sizes. Based on the homoeologous AS triad list generated, PSI for each event triad from A, B and D genomes was extracted separately, and was then treated as the same element for the downstream PCA. PCA was performed using the PCA function in R FactoMineR package. Bi‐plots of PC1 & PC2 and PC3 & PC4 were plotted using R ggplot2 package, and variation explanation percentage for each principal component was calculated by the get_eigenvalue function in R FactoMineR package. 3D‐plot of PCA was generated using R plot3D package. PLS‐DA analysis was performed using R ropls package based on the same data set used for PCA and hierarchical clustering.

### Identification of specific AS events and functional classification

The specificity of AS in different ploidies/stages/tissues was determined, and all replicates of samples were considered. A *k*‐mean cluster was performed to group similar AS events by PSI values, and event clusters that are specific to different ploidies/stages/tissues were identified through a correlation analysis with R cor function using a custom design. p‐values of correlation were computed with R cor.test. All specific AS events were clustered together to plot heat map using R pheatmap package, and *Z*‐score transformation of PSI was performed prior to heatmap plotting.

To determine the functions of genes involved in these specific AS events, we conducted a functional annotation of corresponding genes by performing Gene Ontology (GO) annotations of transcripts and enrichment analysis. GO annotation was conducted through a released exhaustive data set, GOMAP Wheat Reference Sequences 1.1 from Iowa State University (https://dill‐picl.org/projects/gomap/gomap‐datasets/), and only biological process terms were applied. GO enrichments of each gene category containing specific AS events were performed using the R clusterProfiler package. All expressed genes in different wheat species were used as background. REViGO analysis (http://revigo.irb.hr) was used to slim the enriched GO terms based on the ‘medium similarity’ parameter.

### Correlation coefficient analysis of AS event

Dynamic transcriptional expression patterns from early to late stages of embryonic development were used as a gene or transcript characteristic to determine the correlation coefficient between homoeologous genes or homoeologous AS events. Pairwise comparisons of these gene and transcript characteristics among three members of a triad were performed through (i) Pearson's correlation coefficient (*r*) using TPM values of gene expression and (ii) Spearman's rank correlation coefficient (ρ) using PSI, respectively, using cor function in R base package. *P*‐values were calculated using the corr.test function in R psych package. Conserved correlation of gene expression patterns was defined as *r* > 0.8 in all three pairwise correlation analysis, while unconserved AS events were defined as ρ < 0.5 existing in at least one pairwise correlation analysis. Correlation analysis of TPM/PSI matrices was performed using the mapply function in R base package.

### Generation of phylotrees and tree length computation

The divergence of transcripts in each subgenome of two hexaploid wheat cultivars was computed through neighbour‐joining trees based on matrices of pairwise subgenomes distance. The distance between two subgenomes was calculated through hierarchical clustering performed using R ape package, and R dist function was used to determine the final distance based on the pairwise Pearson's correlation coefficient (*r*) of the AS triads among three subgenomes. Based on the PSI values of these AS triads, we bootstrapped 100 replicate trees for three subgenomes in AC and CS for tree length calculation. We then compared the distributions of bootstrapped total tree lengths between subgenomes, evaluating the significance of differences using Duncan's tests, and the results were shown in violin plots using the R ggviolin function in ggpubr package.

### Genomic phylostratigraphy and polyploidization analysis

Phylostratum (PS) in evolutionary transcriptome analysis was determined using a method reported previously (Quint *et al.,* 2012). The PS level of each transcript was determined based on a database (http://msbi.ipb‐halle.de/download/phyloBlastDB_Drost_Gabel_Grosse_Quint.fa.tbz) including thousands of genomes across species in 14 PS levels using a custom script (https://github.com/AlexGa/Phylostratigraphy). Distribution of AS event number per gene was calculated in each PS level and compared.

Analysis of AS triads during polyploidization first focused on the diploid species, in order to select event triads with at least 50 PSI difference between DV and SP (for tetraploid polyploidization) or among DV, SP and TA (for hexaploid polyploidization). Then, the PSI differences between diploid and tetraploid/hexaploid in corresponding subgenomes were calculated and the AS events were defined to be influenced by polyploidization if the PSI difference is above 20. Finally, the AS triads influenced by polyploidization were clustered for heatmap generation and functional prediction.

### Cost‐efficient RNAseq validation of splice junction sites and expression of homoeologous transcripts using a droplet digital PCR (ddPCR) assay

We performed two strategies for ddPCR assays to validate AS events. One used primers with at least two close SNPs or one indel at the 3′ end and two probes targeting the different transcripts across AS junction sites. The other strategy was to use one primer spanning the AS junction site and two probes targeting the region in the PCR products with 2–3 SNPs among homoeologous genes in three subgenomes. Comparing these two strategies, the latter showed more advantages. The first strategy required a high density of SNPs close to AS junctions, limiting the options for validation. Furthermore, the first strategy required three reactions to detect a PSI for one AS triad, while we optimized the second strategy so that only two reactions are required for a triad's PSI detection, resulting in cost‐efficiency (Figure 8a). For the second strategy, successful primer and probe designs entail the following criteria:

1. An AS junction‐spanning feature should be contained in primers. Then, two pairs of primers can separately amplify the two transcripts of the AS event. The junction site should be close to 3′ end in primer design.
2. Primer sequences should be strictly conserved among three homoeologous genes. In other words, the primer fragments require no SNP/indels in a triad from three subgenomes.
3. To distinguish three homoeologous genes, probes should target a region, where in comparison with the first gene in a triad, the second and third genes share an SNP and the third gene possess 1–2 extra SNPs. If the third gene has two extra SNPs, these two SNPs should be at least 8 base pairs (bp) away from each other and located on both sides of shared SNP. The best position for shared SNP is in the middle of the probe. Two fluorescent reporters are added to the probes targeting the first and third homoeologous genes.
4. The final concentration of transcripts should be optimized to less than 100 copies/μL for accurate calculation in this method.
5. Other common rules for primer and probe design should also be satisfied, including 35%–65% GC content, complementarity and secondary structure, probes with 6–10 °C higher Tm than primers, more C than G, avoiding G at 5′ end, avoiding G and C repeats (more than three) in probes.

In the present study, probes were 5′‐labelled with 6‐carboxyfluorescein (6‐FAM) or 6‐carboxy‐2, 4, 4, 5, 7, 7 hexachlorofluorescein succinimidyl ester (6‐HEX) as the reporter and ZEN/Iowa Black FQ as the 3′‐labelled double quenchers. Ideally, the first and third homoeologs can be perfectly captured by these two fluorescent probes, while the second homoeolog with 1–2 SNPs to either probes will be detected in 2‐D mode as a weak signal cluster between two strong signal clusters (Figure 8b). This method with two probes detecting three homoeologous genes in one reaction was further verified through three reactions using three specific probes targeting three homoeologs, respectively. Primer and probe sequences for each target and reference genes are provided in Table [Link], [Link].

RNA extracted using methods described above was treated with DNase I (Thermo Fisher Scientific, San Jose, CA, USA) and then reverse transcribed using the SuperScript IV VILO system (Thermo Fisher Scientific, San Jose, CA, USA) according to the manufacturer's instructions. Transcript abundance was measured using the Bio‐Rad QX200 ddPCR System (Bio‐Rad, Richmond, CA, USA). In brief, each 20 μL 1 x ddPCR SuperMix for probes reaction mixture (no dUTPs, Bio‐Rad) containing cDNA templates, forward and reverse primers and two probes with optimized concentration was mixed with 70 μL of Droplet Generation oil for Probes (Bio‐Rad) in a DG8 Cartridge (Bio‐Rad). The cartridge was covered with a DG8 gasket and loaded into the QX200 Droplet Generator (Bio‐Rad) to generate PCR droplets. From each droplet mix, 40 μL was transferred to a 96‐well PCR plate (Bio‐Rad) and sealed using PX1™ PCR plate Sealer (Bio‐Rad). PCR thermal cycling was optimized, and the amplification signals were read using the QX200™ Droplet Reader and analysed using QuantaSoft software (Bio‐Rad) in 2‐D mode.

## Conflicts of Interest

The authors declare no competing interests.

## Author contributions

DX and RD conceived of and coordinated the study. PG, TDQ, CZ, LQ and DX performed experiments. PG and CZ performed bioinformatic analyses. PG, TDQ, DX and CSG performed data analysis, prepared figures and wrote the manuscript. KTN, QL, AGS, LVK, JZ, ASNR, YW, CP, NP and RD contributed to manuscript preparation. All authors read and approved the final manuscript.

## Data Access

All raw and processed sequencing data generated in this study have been submitted to the NCBI Gene Expression Omnibus (GEO; https://www.ncbi.nlm.nih.gov/geo/) under accession number ‘GSE129695’ (AC, ST, DV, SP, TA) and ‘GSE153420’ (CS, CM).

## Supporting information

Supplementary Material
**Figure S1** Pipeline for analysis of evolution of splicing in polyploid plants, using embryos as an example.
**Figure S2** Statistics of AS events.
**Figure S3** Statistics of AS events at the subgenomes, stages and tissues level.
**Figure S4** Principal component analysis (PCA) of gene expression and alternative splicing regulation in A, B, D subgenomes from different wheat varieties.
**Figure S5** Heatmap of correlations between co‐expressed AS event clusters and ploidies.
**Figure S6** Distribution of AS event types for ploidy‐specific events.
**Figure S7** Characterization of AS triads with balanced gene‐level expression and unbalanced PSI ratio during embryogenesis.
**Figure S8** Examples of dynamic AS with stable GE during embryogenesis.
**Figure S9** Portion of the variance (PoV) explained by each of the principal components in PCA based on conserved AS triads.
**Figure S10** Phylogenetic trees built using AS triads in Hexaploid wheats (AC and CS) during embryogenesis.
**Table S1** Seven wheat cultivars and their putative diploid ancestors and sampling stages/tissues used in this study.
**Table S2** Transcript isoforms identified in this study.
**Table S3** Statistical analysis of AS events in each gene locus across seven wheat cultivars and their putative diploid ancestors.
**Table S4** Portion of the variance (PoV) explained by each of the principal components in PCA from Figure 3 and Figure S4.
**Table S5** Primers and probes used in this study.

**Data S1** PSI of AS events in AC Barrie (AC).

**Data S2** PSI of AS events in Chinese Spring (CS).

**Data S3** PSI of AS events in Commander (CM).

**Data S4** PSI of AS events in Strong Field (ST).

**Data S5** PSI of AS events in DV92 (DV).

**Data S6** PSI of AS events in TA2780 (SP).

**Data S7** PSI of AS events in TA101132 (TA).

**Data S8** List of AS events and GO enrichment results in different categories.

**Data S9** Clusters of AS events identified in A, B and D subgenomes of polyploid wheats and their putative ancestors.

**Data S10** Conserved AS triads identified using chain files from A, B and D subgenomes of Chinese Spring.

**Data S11** AS triads exhibiting balanced gene level expression with biased splicing from ten stages/tissues.

**Data S12** Correlation analysis of AS triads.

**Data S13** Phylostratum (PS) levels of each transcript in AS events.

**Data S14** AS triads affect by polyploidization in wheat.
