# Supplementary material for: Alternative splicing dynamics and evolutionary divergence during embryogenesis in wheat species
Source: Plant Biotechnol J. 2021 Mar 24;19(8):1624–43. doi: 10.1111/pbi.13579 (PMC8384600; doi:10.1111/pbi.13579)
Supplement: Supplementary file 1 — Supplementary Material Figure S1 Pipeline for analysis of evolution of splicing in polyploid plants, using embryos as an example. Figure S2 Statistics of AS events. Figure S3 Statistics of AS events at the subgenomes, stages and tissues level. Figure S4 Principal component analysis (PCA) of gene expression and alternative splicing regulation in A, B, D subgenomes from different wheat varieties. Figure S5 Heatmap of correlations between co‐expressed AS event clusters and ploidies. Figure S6 Distribution of AS event types for ploidy‐specific events. Figure S7 Characterization of AS triads with balanced gene‐level expression and unbalanced PSI ratio during embryogenesis. Figure S8 Examples of dynamic AS with stable GE during embryogenesis. Figure S9 Portion of the variance (PoV) explained by each of the principal components in PCA based on conserved AS triads. Figure S10 Phylogenetic trees built using AS triads in Hexaploid wheats (AC and CS) during embryogenesis. Table S1 Seven wheat cultivars and their putative diploid ancestors and sampling stages/tissues used in this study. Table S2 Transcript isoforms identified in this study. Table S3 Statistical analysis of AS events in each gene locus across seven wheat cultivars and their putative diploid ancestors. Table S4 Portion of the variance (PoV) explained by each of the principal components in PCA from Figure 3 and Figure S4. Table S5 Primers and probes used in this study. [file PBI-19-1624-s013.pdf]

Supplemental Table

Genus Species		*Triticum monococcum*	*Aegilops speltoides*	*Aegilops tauschii*	*Triticum turgidum*		*Triticum aestivum*	
**Variety**		DV92	TA2780	TA101132	Strong Field	CDC commander	Chinese Spring	AC Barrie
**Abbreviated name**		DV	SP	TA	SF	CM	CS	AC
**Genome**		AA	BB	DD	AABB	AABB	AABBDD	AABBDD
**Chromosome Number**		14	14	14	28	28	42	42
**Ploidy**		Diploid	Diploid	Diploid	Tetraploid	Tetraploid	Hexaploid	Hexaploid
**Definition of stages**		**hour (HAF) or day (DAF) after fertilization**						
**two cell embryo**	**E01**	24-30HAF	24-30HAF	24-30HAF	24-36HAF	24-36HAF	24-36HAF	24-36HAF
**pre-embryo**	**E02**	72-78HAF	72-78HAF	72-78HAF	72-84 HAF	72-84 HAF	72-84HAF	72-84HAF
**transition embryo**	**E03**	7DAF	7DAF	7DAF	8DAF	8DAF	9DAF	9DAF
**leaf early embryo**	**E04**	10DAF	10DAF	10DAF	11DAF	11DAF	12DAF	12DAF
**leaf middle embryo**	**E05**	15DAF	15DAF	15DAF	16DAF	16DAF	17DAF	17DAF
**leaf late embryo**	**E06**	20DAF	20DAF	20DAF	22DAF	22DAF	24DAF	24DAF
**mature embryo**	**E07**	26DAF	26DAF	26DAF	31DAF	31DAF	34DAF	34DAF
**transition stage endosperm**	**E08**	7DAF	7DAF	7DAF	8DAF	8DAF	9DAF	9DAF
**leaf late stage endosperm**	**E09**	20DAF	20DAF	20DAF	22DAF	22DAF	24DAF	24DAF
**leaf early stage pericarp**	**E10**	10DAF	10DAF	10DAF	11DAF	11DAF	12DAF	12DAF

Supplemental Table 1. Seven wheat cultivars and their putative diploid ancestors and sampling stages/tissues used in this study. Genus names, selected varieties, ploidy information, abbreviations used in this study and sampling times are indicated.

	gene loci	transcripts	multi-exon transcripts	multi-transcript loci	transcripts per locus
IWGSC RefSeq v2.0	113,001	138,666	104,387	16,789	1.2
Hexaploid (AC, CS)	103,309	223,873	194,562	44,205	2.2
Tetraploid (CM, ST)	69,645	154,535	134,825	30,122	2.2
Diploid (DV)	34,804	75,691	65,911	14,711	2.2
Diploid (SP)	34,841	78,844	68,914	15,411	2.3
Diploid (TA)	33,664	69,338	59,737	14,083	2.1

Supplemental Table 2. Transcript isoforms identified in this study. The numbers of gene loci including multi-transcript loci and transcripts including multi-exon transcripts were shown. Average transcripts per locus were calculated.

AS event(s) per gene	No. of AS genes	No. of AS events	% of AS genes
1	13,152	13,152	46.33
2	5,766	11,532	20.31
3	3,478	10,434	12.25
4	1,970	7,880	6.94
5	1,150	5,750	4.05
6	807	4,842	2.84
7	563	3,941	1.98
8	360	2,880	1.27
9	261	2,349	0.92
10	198	1,980	0.70
11	155	1,705	0.55
12	142	1,704	0.50
13	96	1,248	0.34
14	77	1,078	0.27
15	65	975	0.23
16	41	656	0.14
17	28	476	0.10
18	29	522	0.10
19	27	513	0.10
20	24	480	0.08

Supplemental Table 3. Statistical analysis of AS events in each gene locus across seven wheat cultivars and their putative diploid ancestors. The distribution of AS events containing genes by their AS event numbers. Only AS events with 5<PSI<95 in at least one sample were counted.

	PSI				GE			
subgenomes	A	B	D	AB	A	B	D	AB
PCA figures	Fig. 3A	Sup. Fig. 4A	Sup. Fig. 4C	Sup. Fig. 4E	Fig. 3B	Sup. Fig. 4B	Sup. Fig. 4D	Sup. Fig. 4F
PC1	15.4%	28.2%	27.5%	13.2%	17.0%	16.5%	19.1%	19.9%
PC2	9.6%	6.4%	8.4%	7.2%	13.4%	13.8%	14.1%	15.9%
PC3	6.2%	5.0%	6.1%	4.4%	11.8%	12.0%	12.0%	13.1%
PC4	3.5%	3.4%	4.5%	3.9%	10.5%	10.6%	10.4%	7.7%
PC5	2.7%	3.0%	3.4%	3.3%	5.8%	5.8%	5.9%	7.0%
PC6	2.4%	2.2%	2.9%	2.8%	5.6%	5.1%	4.6%	4.4%
PC7	2.1%	1.9%	2.6%	2.7%	3.2%	3.5%	4.1%	3.4%
PC8	1.9%	1.8%	2.4%	2.3%	2.9%	2.5%	3.7%	2.8%
PC9	1.7%	1.6%	2.2%	2.1%	2.5%	2.2%	2.6%	2.5%
PC10	1.7%	1.5%	2.1%	1.9%	2.2%	2.2%	2.4%	2.1%

Supplemental Table 4. **Portion of the variance (PoV) explained by each of the principal components in PCA from Figure 3 and Supplemental Figure 4.** PoV from PC1-PC10 was listed for each figure, the information about subgenomes, corresponding figures for both PSI and GE categories were provided.

No.	Orientation	Target gene	Sequence (5' to 3')
DG322	Forward primerF1	MSTRG.214389, MSTRG.231998, MSTRG.249062	AGGGATCAGATACCATCTCGAAC
DG323	Reverse primerR1	MSTRG.214389, MSTRG.231998, MSTRG.249062	ACCCACATTACTTCCATATAATTGC
DG324	Probe in A subgenome	MSTRG.214389/ TraesCS5A02G074900	FAM-CCGTGTTCACGCAGAACCTGCA-ZEN/IABkFQ
DG325	Probe in B subgenome	MSTRG.231998/ TraesCS5B02G081300	HEX-CCGTGTGCACGCAGAACCTGCA-ZEN/IABkFQ
DG326	Probe in C subgenome	MSTRG.249062/ TraesCS5D02G088400	FAM-CCGTGTGCACACAGAACCTGCA-ZEN/IABkFQ
DG327	Reverse primerR2	MSTRG.214389, MSTRG.231998, MSTRG.249062	TCTACATCTTCATCGACCTTCCAT
DG363	Probe in C subgenome	MSTRG.249062/ TraesCS5D02G088400	HEX-CCGTGTGCACACAGAACCTGCA-ZEN/IABkFQ

Supplemental Table 5. Primers and probes used in this study. The sequence of primers and probes, targeted genes and usages were indicated.

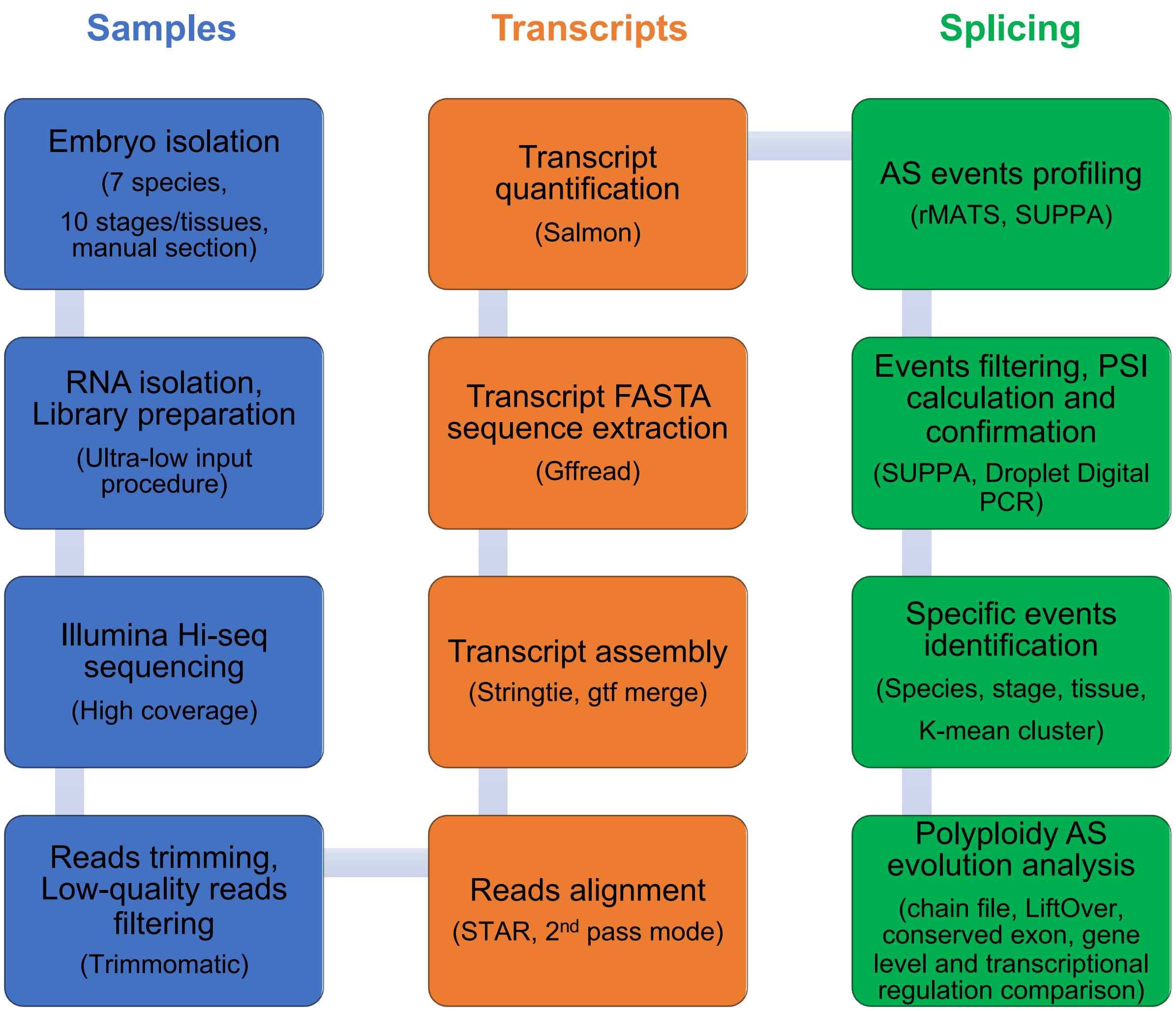

Supplemental Figure 1 Pipeline of polyploid plants splicing evolution analysis using embryo samples as an example. Three major steps focusing on analysis of samples, transcripts and splicing are labeled in different colors. Key words and major analysis tools are indicated in round brackets for each step.

Event structure

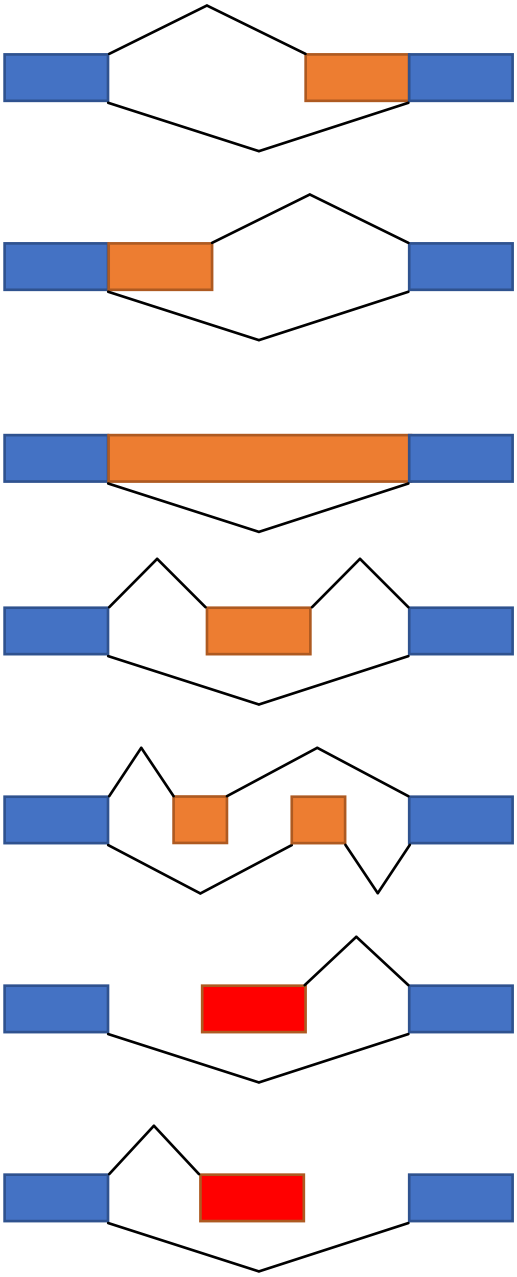

Event	Event description	Ratio
A3	**Alternative 3' splice-site**	**33%**
A5	**Alternative 5' splice-site**	**19%**
RI	**Retained intron**	**16%**
SE	**Skipping exon**	**13%**
MX	**Mutually exclusive exons**	**0.6%**
AF	**Alternative first exon**	**13%**
AL	**Alternative last exon**	**5%**

Supplemental Figure 2 Statistics of AS events. The intron-exon structure of the AS events are illustrated (exons are denoted by boxes and introns are thick black lines, AS regions are shown as orange or red boxes), followed by the event descriptions and the ratio of events (at least 5<PSI<95 in one sample) found in all samples in this study.

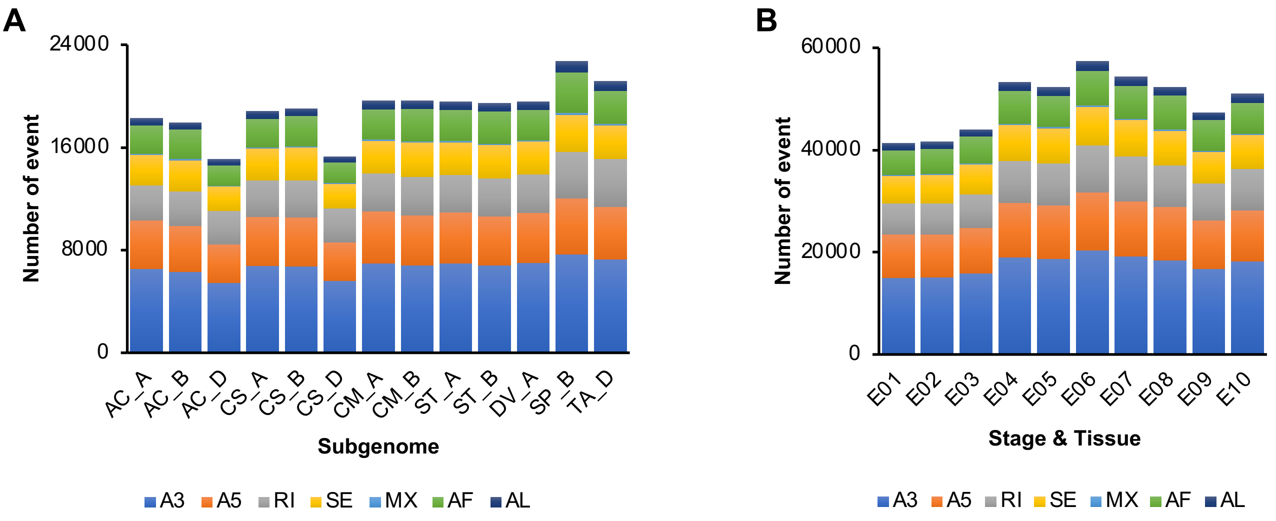

Supplemental Figure 3 Statistics of AS events in subgenome, stage and tissue level. (A) Distribution of seven types of AS events in different subgenomes. First two letters in X-axis indicate species, the third letter followed by underlines indicates subgenomes. E.g., AC_A means A subgenome of AC Barrie. **(B)** Distribution of seven types of AS events in different stage and tissues.

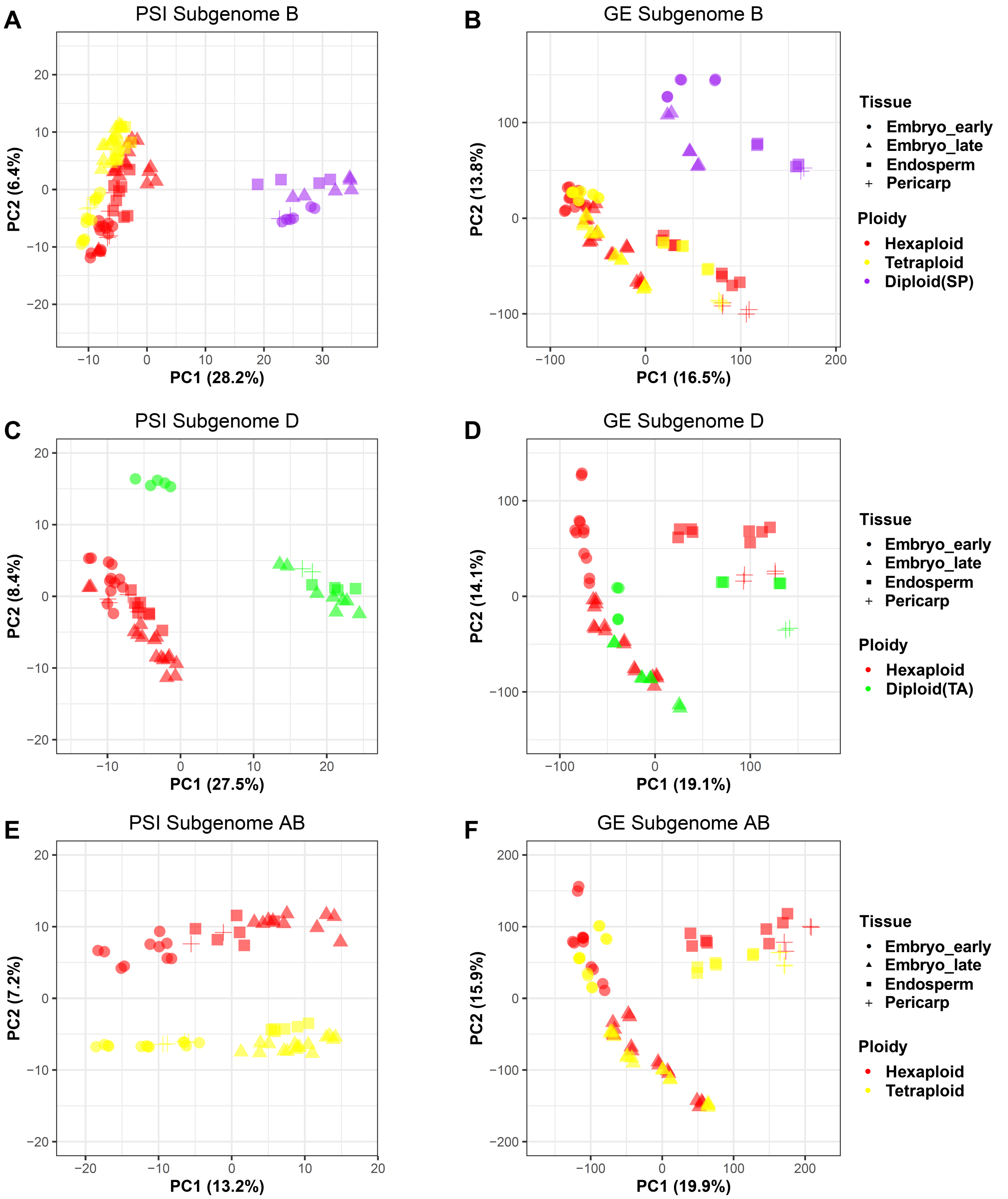

Supplemental Figure 4 Principal component analysis (PCA) of gene and transcriptional level regulation in different species. PCA based on PSI from different species in B subgenome **(A)**, D subgenome **(C)** and both A and B subgenomes **(E)** were plotted on the left. PCA based on gene expression (GE) from different species in B subgenome **(B)**, D subgenome **(D)** and both A and B subgenomes **(F)** were plotted on the right. X-axis and Y-axis indicate PC1 and PC2, respectively. Different stages and tissues are labeled with different shapes, different ploidies are labeled with different colors.

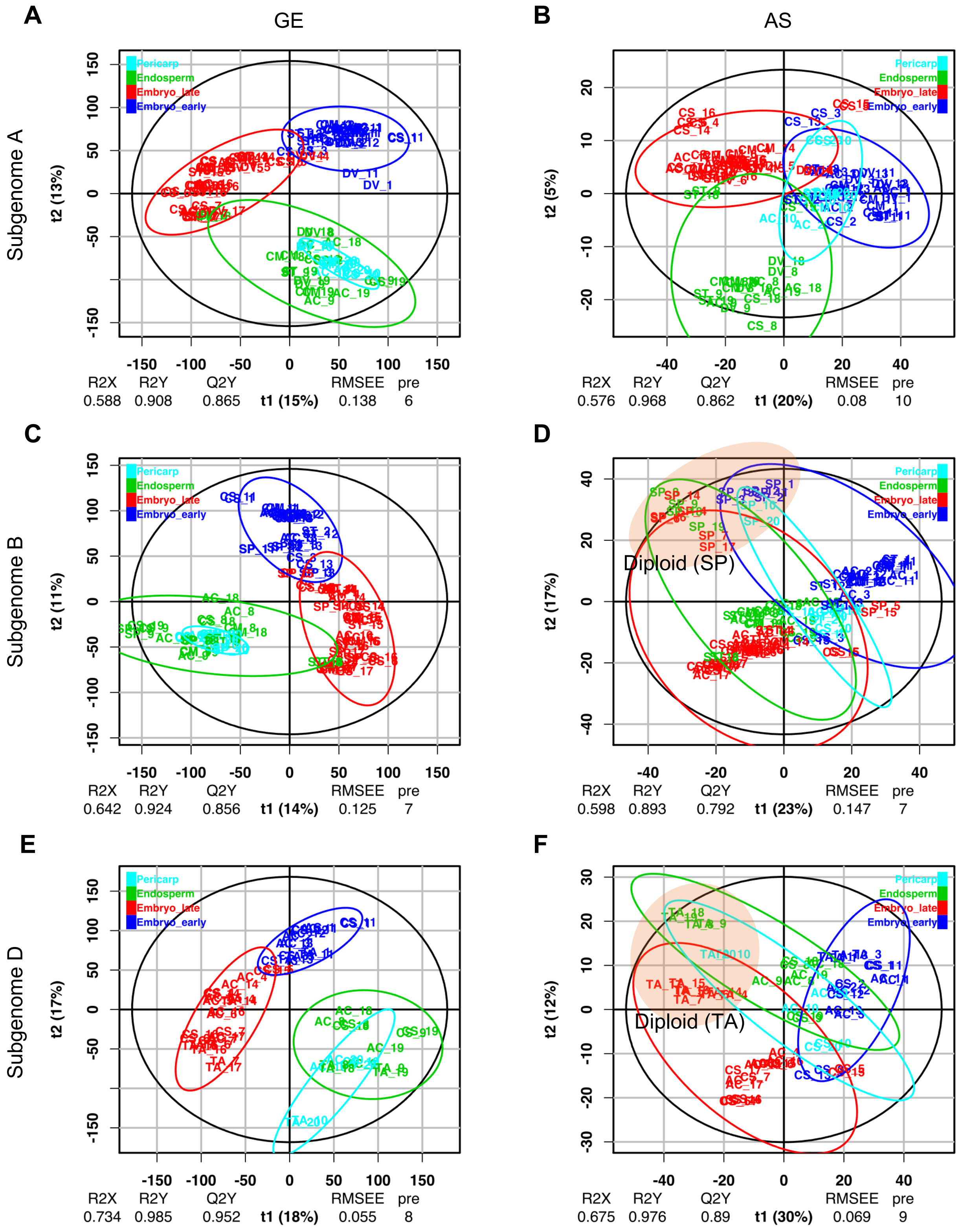

Supplemental Figure 5 Regression analysis of AS and GE in polyploid wheat and diploid ancestral species from different stages and tissues during grain development using partial least squares discriminant analysis (PLS-DA). A-F. PLS-DA of GE (**A, C, E**) and AS (**B, D, F**) by forcing the separation among different embryo developmental stages and other tissues in subgenome A (**A, B**), B (**C, D**) and D (**E, F**)**.** Score plots of PLS-DA models used same samples for PCA plot in Figure 3 and Supplemental Figure 4. Samples from the same developmental stages and tissues are labeled with same colors, as shown in the top left. Transparent ovals are used to group individuals in the same stages/tissues. Solid ovals in **D** and **F** are used to group the samples from diploid SP and TA, respectively.

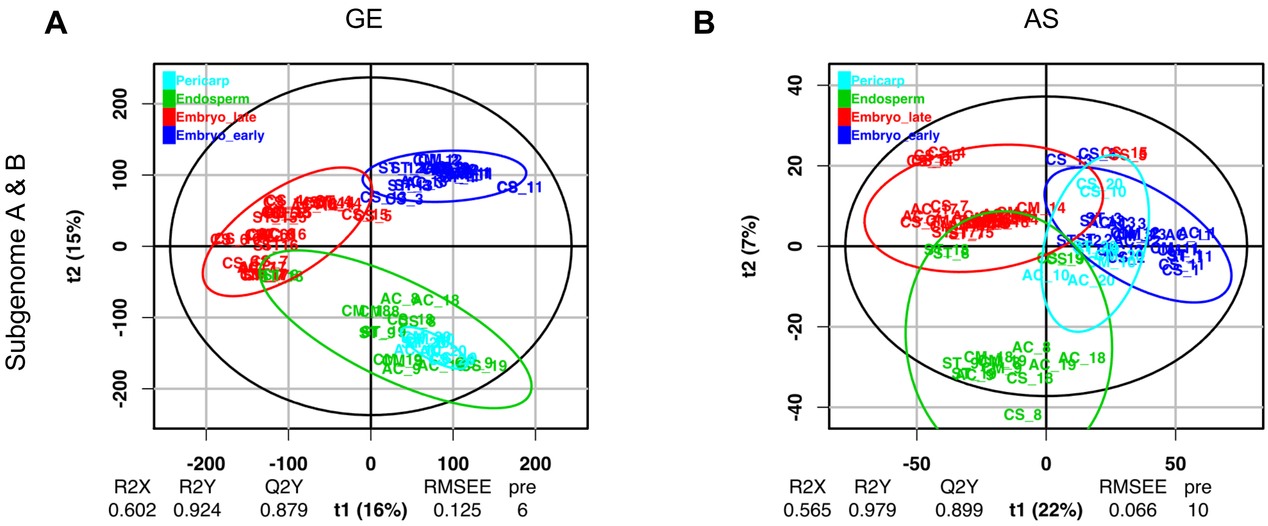

Supplemental Figure 6 Regression analysis of AS and GE in hexaploid and tetraploid species from different stages and tissues during grain development using partial least squares discriminant analysis (PLS-DA). A-B. PLS-DA of GE (**A**) and AS (**B**) by forcing the separation among different embryo developmental stages and other tissues. Score plots of PLS-DA models used same samples for PCA plot in Supplemental Figure 4E and F. Samples from the same developmental stages and tissues are labeled with same colors, as shown in the top left. Transparent ovals are used to group individuals in the same stages/tissues.

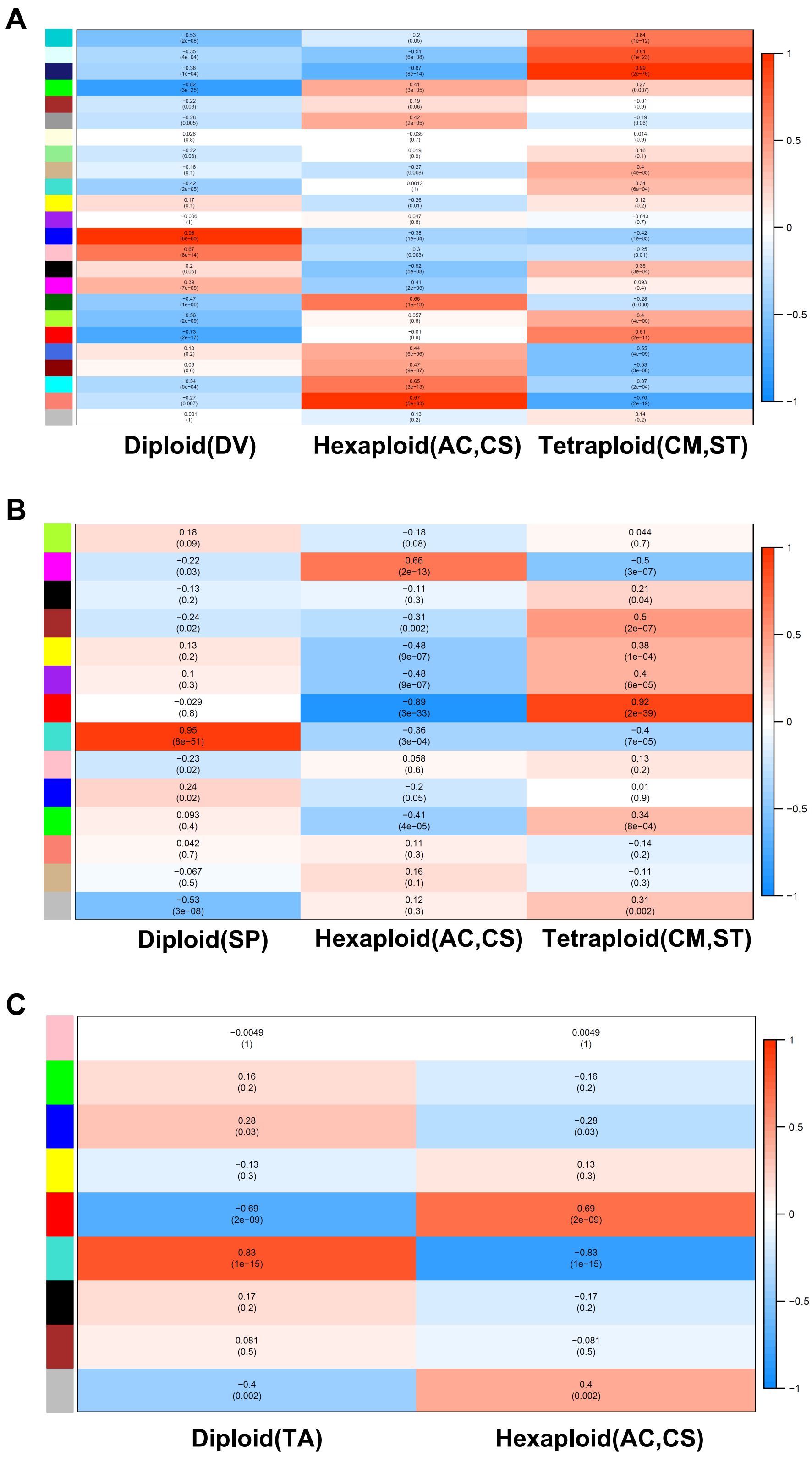

Supplemental Figure 7 Heatmap of correlations between co-expressed AS clusters and ploidies. A. AS in A subgenome; **B.** AS in B subgenome; **C.** AS in C subgenome. The color scheme, from red through white to green, indicates the levels of correlation, from high to low. The Pearson correlation value (r) and p-values are indicated in boxes for each cluster. The ploidy-specific modules are identified by r > 0.8, p-value < 0.001.

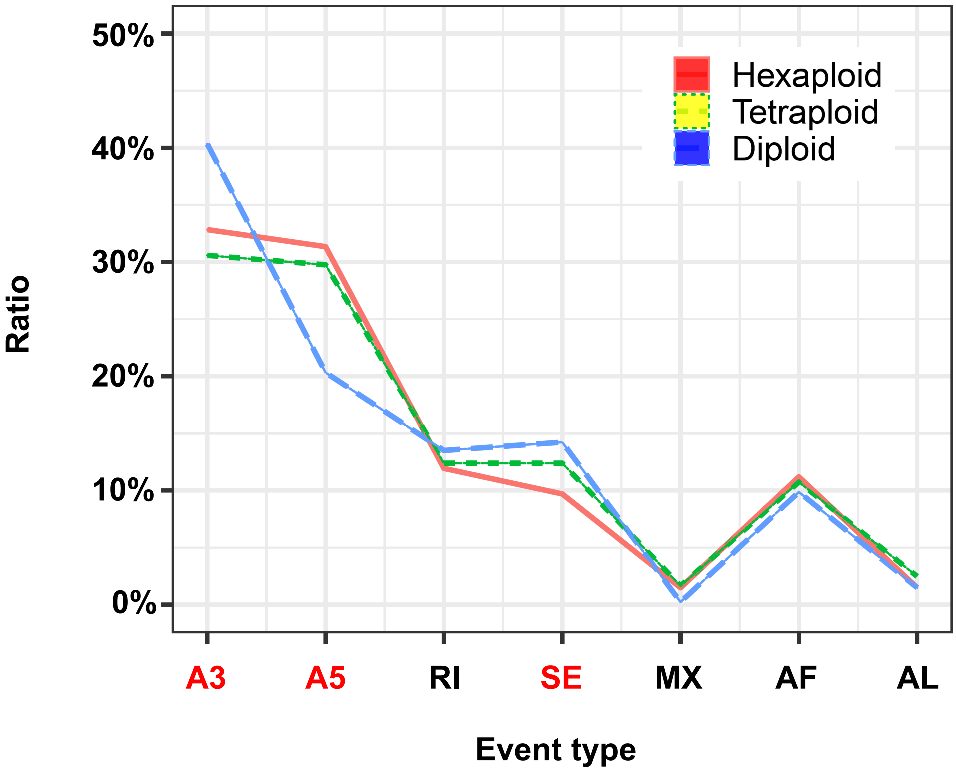

Supplemental Figure 8 Distribution of AS types for ploidy-specific events. X-axis, seven types of AS events, red color labels indicate significant difference among different ploidy-specific event clusters. Y-axis, event type ratios.

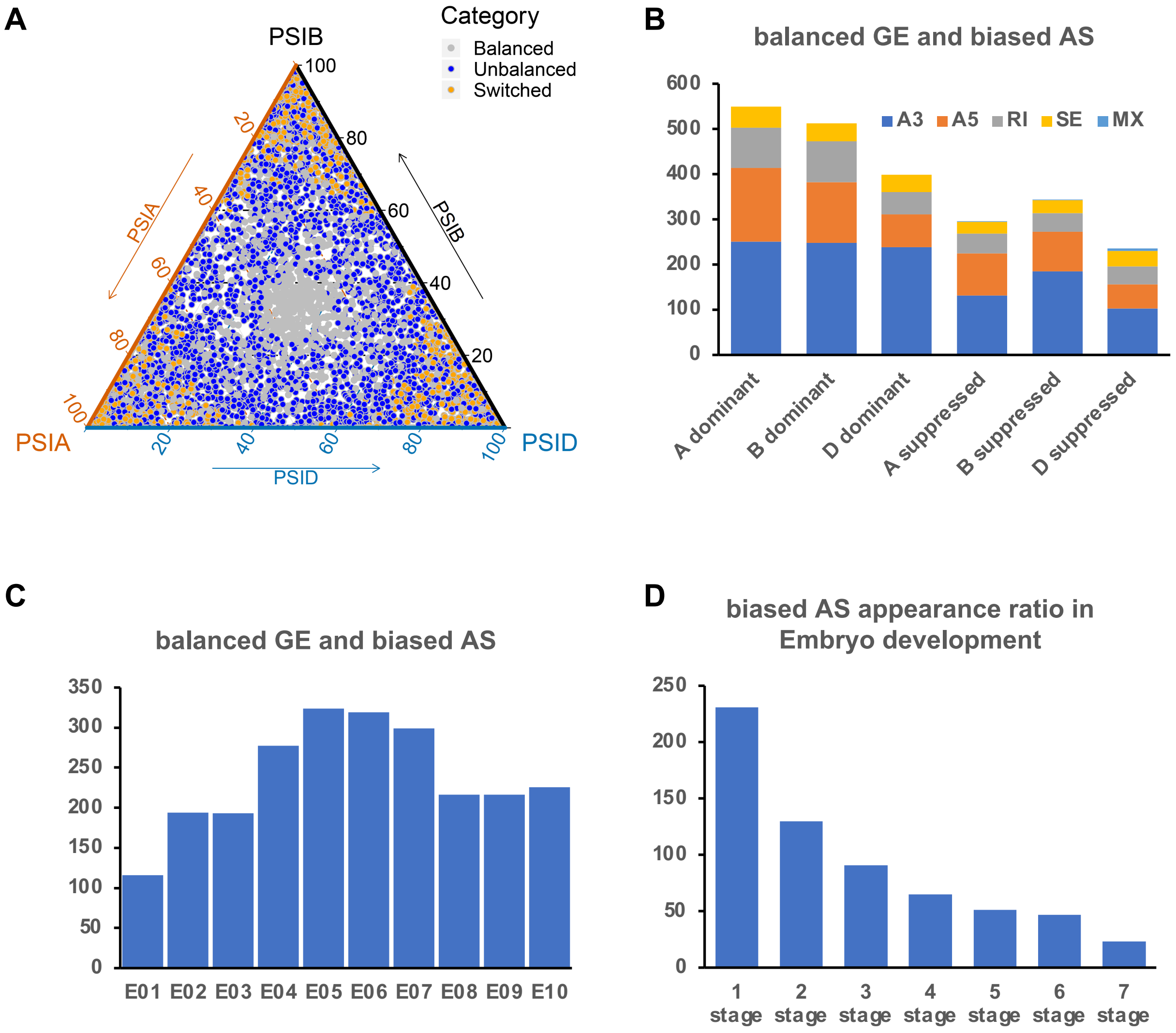

Supplemental Figure 9 Characterization of AS triads with balanced gene level expression and unbalanced PSI ratio during embryogenesis. (A) Ternplot of three categories AS events (Balanced, Unbalanced and Switched) in 1950 AS conserved triads (only AS events with TPM > 1 in all three members (A,B,D) of one triad were plotted). **(B)** Number of six categories of biased AS events with balanced gene level expression (GE) during embryogenesis. **(C)** Distribution of biased AS events with balanced GE in different stages and tissues. **(D)** Number of biased AS events with balanced GE appearing in multiple stages during embryogenesis.

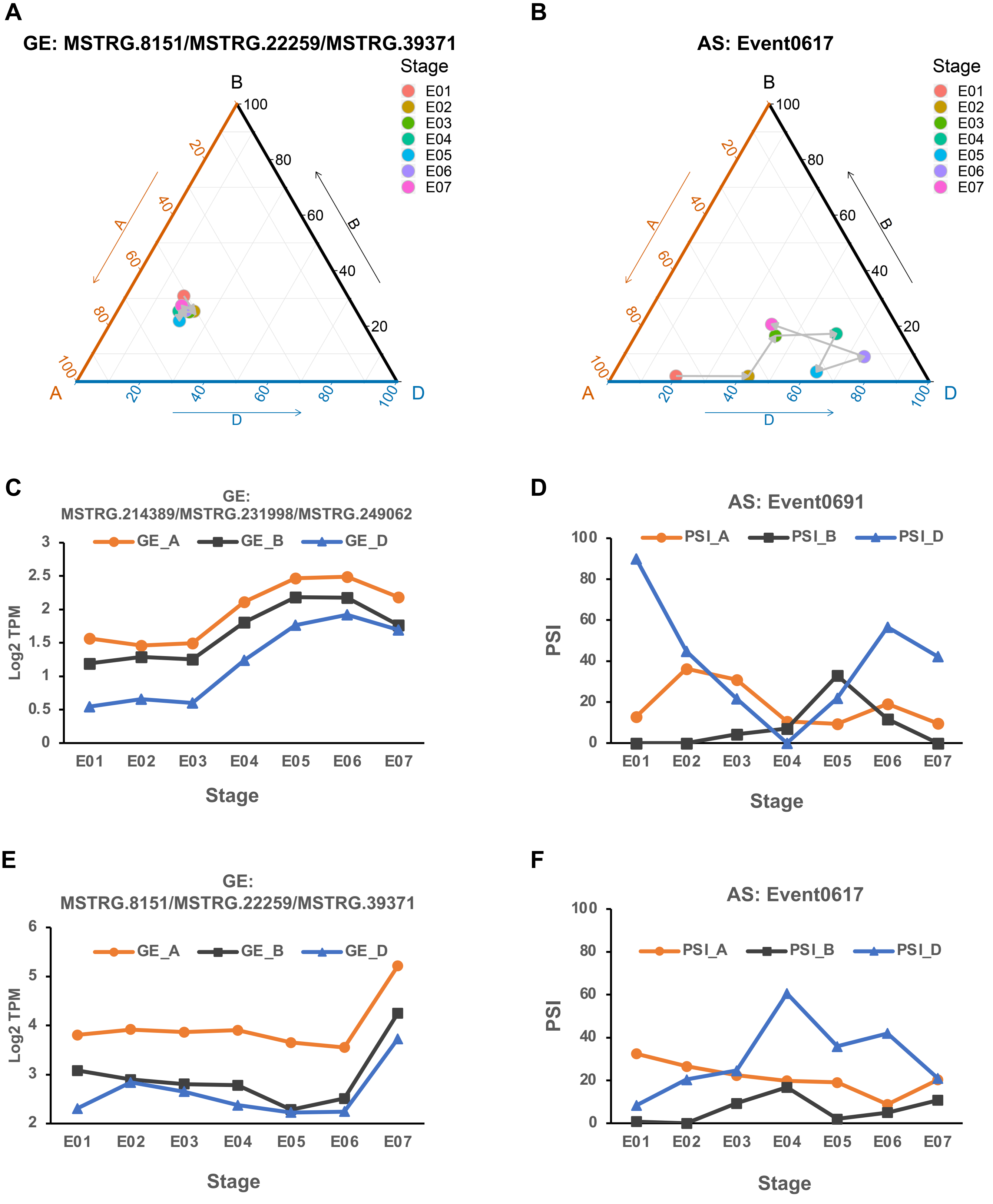

Supplemental Figure 10 Examples of dynamic AS with stable GE during embryogenesis. (A) Ternplot of gene expression ratio related to AS triad Event0617. **(B)** Ternplot of PSI ratio related to AS triad Event0617. The developmental process from E1 to E7 during embryogenesis was following the order labeled by arrows. **(C)** gene expression and **(D)** PSI trends related to AS triad Event0691 during embryogenesis. **(E)** gene expression and **(F)** PSI trends related to AS triad Event0617 during embryogenesis.

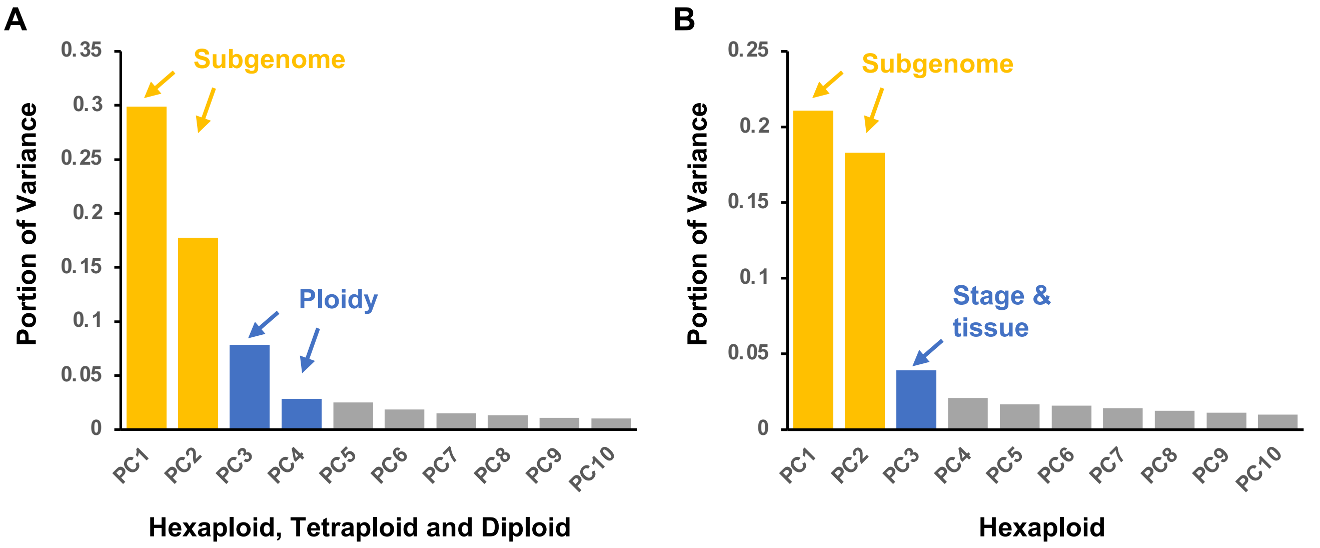

Supplemental Figure 11 Portion of the variance (PoV) explained by each of the principal components in PCA based on conserved AS triads. (A) Barplot of PoV from PCA using conserved triads in Hexaploid(AC, CS), Tetraploid (CM, ST) and Diploid (DV, SP, TA). The dominant effect (subgenome or ploidy) associated with the respective component are indicated in different colors. **(B)** Barplot of PoV from PCA using conserved triads in Hexaploid (AC, CS) only. The dominant effect (subgenome or stage/tissue) associated with the respective component are indicated in different colors.

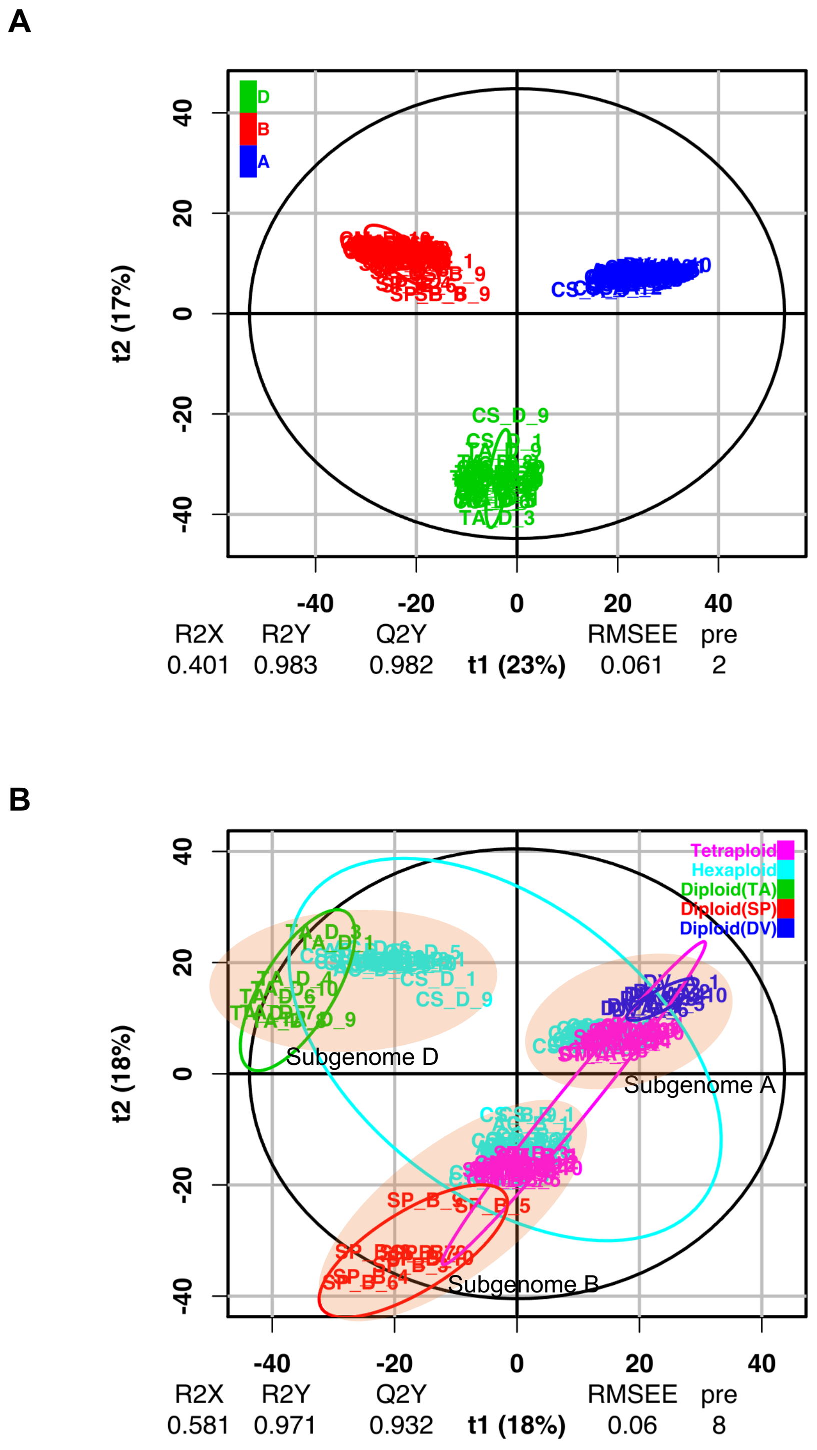

Supplemental Figure 12 Regression analysis of AS in polyploid wheat and diploid ancestral species from different subgenomes and ploidies during grain development using partial least squares discriminant analysis (PLS-DA). A-B. PLS-DA of AS by forcing the separation among different subgenomes (**A**) and different ploidies (**B**). Score plots of PLS-DA models used same samples for PCA plot in Figure 6. Samples from the same subgenomes (**A**) or the same ploidies (**B**) are labeled with same colors, as shown in the top left (**A**) or top right (**B**). Transparent ovals are used to group individuals in the same ploidies. Solid ovals are used to group the samples from the same subgenomes.

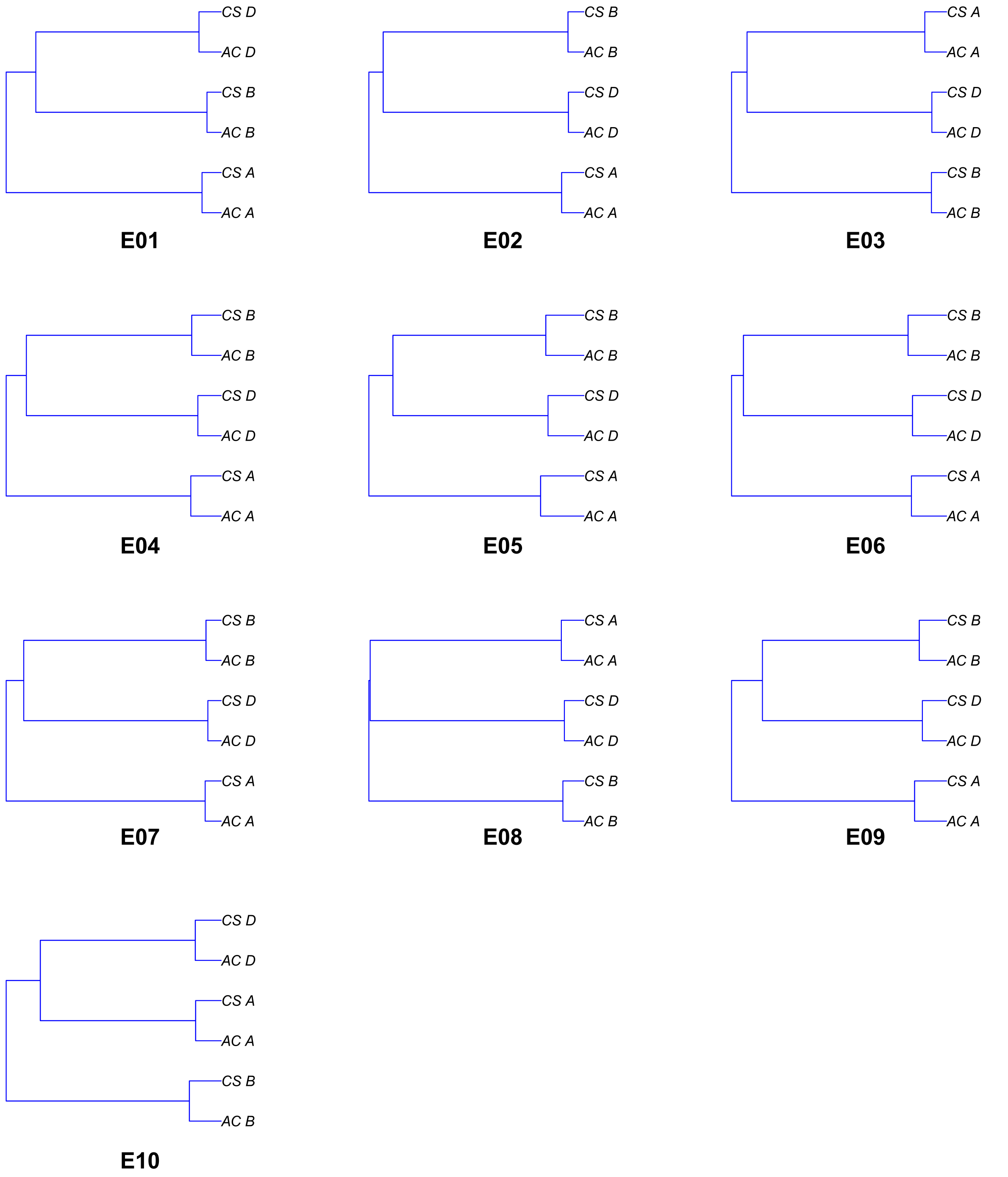

Supplemental Figure 13 Phylogenetic trees built using AS event triads in Hexaploid wheats during embryogenesis. AS neighbor-joining trees based on conserved AS event triads were built with pairwise distance matrices (1 - Pearson correlation of PSI) for each stage/tissue from E01 - E10. For each pair of subgenomes, the PSIs of all AS event triads that are alternative are correlated.
